# Characterization of the targeting signal in mitochondrial β-barrel proteins

**DOI:** 10.1038/ncomms12036

**Published:** 2016-06-27

**Authors:** Tobias Jores, Anna Klinger, Lucia E. Groß, Shin Kawano, Nadine Flinner, Elke Duchardt-Ferner, Jens Wöhnert, Hubert Kalbacher, Toshiya Endo, Enrico Schleiff, Doron Rapaport

**Affiliations:** 1 Interfaculty Institute of Biochemistry, University of Tuebingen, Hoppe-Seyler-Str. 4, 72076 Tuebingen, Germany; 2 Molecular Cell Biology of Plants, Goethe University, Max-von-Laue-Str. 9, 60438 Frankfurt, Germany; 3 Faculty of Life Sciences, Kyoto Sangyo University, Kyoto 603-8555, Japan; 4 Institute for Molecular Biosciences, Center for Biomolecular Magnetic Resonance, Goethe University, Max-von-Laue Str. 9, 60438 Frankfurt, Germany; 5 Cluster of Excellence Frankfurt, Goethe University, Max-von-Laue Str. 9, 60438 Frankfurt, Germany; 6 Buchmann Institute for Molecular Life Sciences, Goethe University, Max-von-Laue Str. 9, 60438 Frankfurt, Germany

## Abstract

Mitochondrial β-barrel proteins are synthesized on cytosolic ribosomes and must be specifically targeted to the organelle before their integration into the mitochondrial outer membrane. The signal that assures such precise targeting and its recognition by the organelle remained obscure. In the present study we show that a specialized β-hairpin motif is this long searched for signal. We demonstrate that a synthetic β-hairpin peptide competes with the import of mitochondrial β-barrel proteins and that proteins harbouring a β-hairpin peptide fused to passenger domains are targeted to mitochondria. Furthermore, a β-hairpin motif from mitochondrial proteins targets chloroplast β-barrel proteins to mitochondria. The mitochondrial targeting depends on the hydrophobicity of the β-hairpin motif. Finally, this motif interacts with the mitochondrial import receptor Tom20. Collectively, we reveal that β-barrel proteins are targeted to mitochondria by a dedicated β-hairpin element, and this motif is recognized at the organelle surface by the outer membrane translocase.

## Abstract (web summary)

Mitochondrial β-barrel proteins are synthesized in the cytosol before being targeted to the organelle. Here, Jores *et al.* show that a specialized hydrophobic β-hairpin motif is the previously undefined targeting sequence and is recognized by the mitochondrial outer membrane translocase.

## Graphical abstract

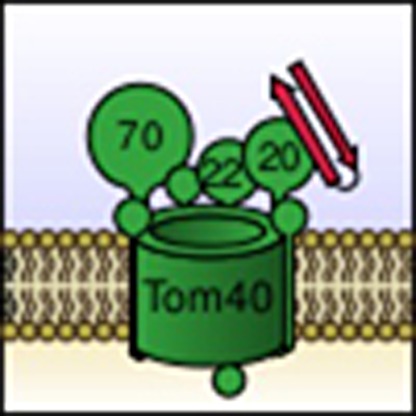

Membrane-embedded β-barrel proteins are found in both prokaryotic and eukaryotic organisms. In prokaryotes such proteins are found in the outer membrane (OM) of Gram-negative bacteria, whereas in eukaryotes they reside exclusively in the OM of mitochondria and chloroplasts. Bacterial β-barrel proteins are synthesized in the cytoplasm with an N-terminal signal sequence for transport across the inner membrane into the periplasm via the SEC system^1^. Their integration into the OM is facilitated by the β-barrel assembly machinery (Bam), the central component of which is the essential protein BamA (Omp85/YaeT)^2^,^3^.

In mitochondria, precursors of β-barrel proteins are synthesized in the cytosol without a cleavable signal sequence. Upon their synthesis, these precursors are translocated from the cytosol into the intermembrane space (IMS) via the translocase of the outer membrane (TOM complex)^4^,^5^. Their subsequent assembly into the OM depends on a dedicated translocase, the topogenesis of OM β-barrel proteins (TOB, also known as sorting and assembly machinery, SAM) complex. The central member of this latter complex is the essential protein Tob55/Sam50 that bears sequence and functional homology to BamA^6^,^7^,^8^. The other two subunits of the TOB complex, Mas37/Sam37 and Tob38/Sam35/Tom38, are peripheral membrane proteins exposed to the cytosol that share no obvious sequence similarity with the accessory lipoproteins of the bacterial Bam complex^9^,^10^,^11^,^12^.

Despite the aforementioned progress in our understanding of the membrane assembly of mitochondrial β-barrel proteins, the mitochondrial targeting signal in such proteins is ill-defined. A conserved linear sequence could not be identified yet and hence it was proposed that the signal is composed by a structural element^13^. However, although cytosolic targeting is a crucial stage in protein biogenesis, neither the character nor the size of such a putative structural signal was identified so far.

In fact, mitochondrial β-barrel proteins in yeast possess a signature (β-signal) that facilitates their intra-mitochondrial sorting^14^. The positioning of this signal in the last strand of the barrel is consistent with the location of a sorting signal in prokaryotic β-barrel proteins^15^. However, this eukaryotic β-signal enhances productive interactions with the TOB complex but does not provide the information required for the initial targeting from the cytosol to the organelle^14^,^16^,^17^,^18^.

In previous efforts to better understand the specific requirements for a mitochondrial targeting signal, bacterial β-barrel proteins were expressed in eukaryotic cells. Such expression resulted in efficient targeting of some of these proteins to mitochondria and their integration into the mitochondrial OM^16^,^17^,^18^,^19^. These studies demonstrate that the bacterial β-barrel proteins possess signals that can target the proteins to mitochondria and can be recognized and processed by the mitochondrial import machinery. Especially informative are our previous observations that mitochondria can recognize and assemble a bacterial trimeric autotransporter β-barrel protein^18^,^19^. The β-barrel anchor of these proteins is formed by a single 12-stranded β-barrel structure to which each monomer contributes four β-strands^20^,^21^. Thus, these findings demonstrate that four β-strands are sufficient for the mitochondria to recognize and assemble a β-barrel protein. However, the precise identity of the signal and its mode of recognition by the organelle import machinery remained unknown.

In the current study we identify a special form of a β-hairpin element as this long searched for targeting signal. A β-hairpin is a characteristic folding motif formed by two β-strands connected by a short loop, and this motif is the smallest building block of all β-barrel proteins^22^. Our *in vitro*, *in organello* and *in vivo* findings demonstrate that a β-hairpin element with one face composed of highly hydrophobic residues is a mitochondrial targeting signal for β-barrel proteins. This signal is recognized at the surface of the organelle by the TOM complex with its import receptor Tom20.

## Results

### Design and structural characterization of β-hairpin peptides

**Figure 1:**
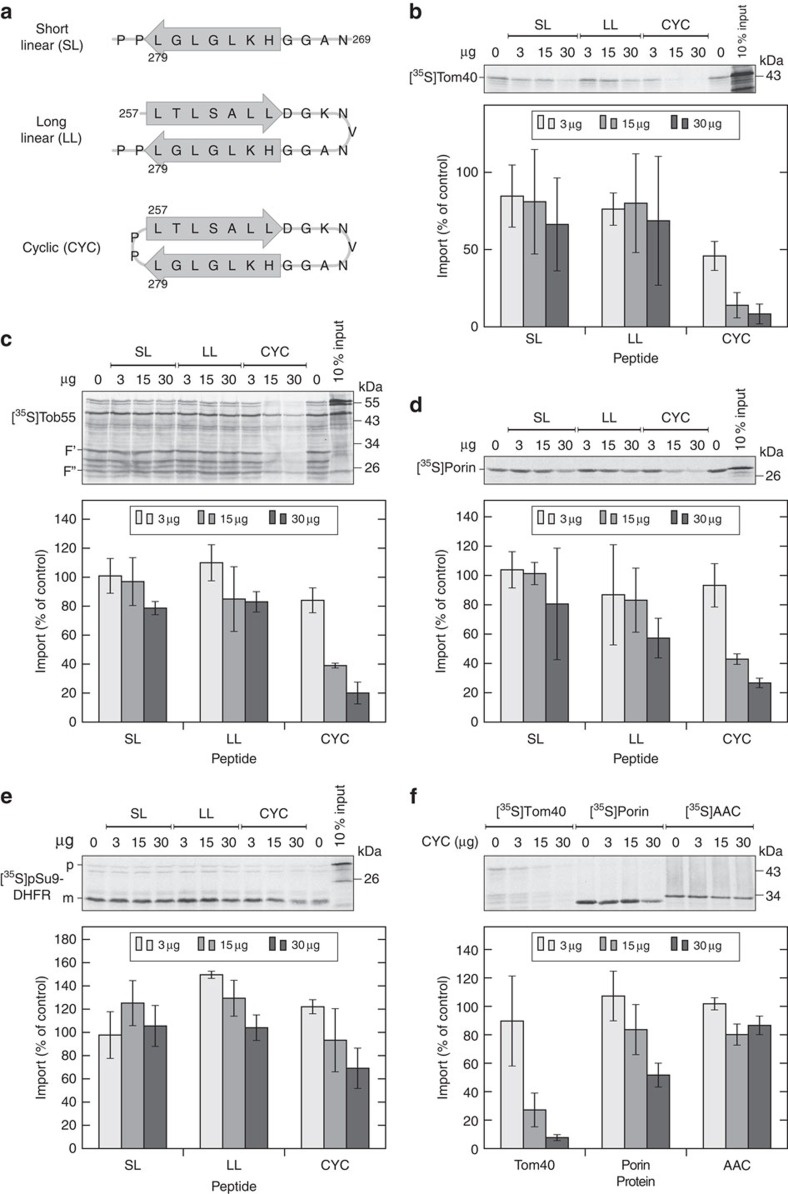
A cyclic β-hairpin peptide inhibits the *in vitro* import of β-barrel proteins. (**a**) Schemes of the three peptides. Grey arrows indicate the residues that form a β-strand in full-length hVDAC1. Residue numbers correspond to the numbering of full-length hVDAC1. (**b**–**e**) Radiolabelled precursor proteins of Tom40 (**b**), Tob55 (**c**), Porin (**d**) or pSu9-DHFR (**e**) were mixed with isolated mitochondria in the presence of the indicated amounts of the short linear (SL), long linear (LL) or cyclic (CYC) peptide. In the end of the import reactions, samples were treated with proteinase K to degrade non-imported molecules. In the case of Tob55, this treatment led to the generation of two protease-protected fragments (F′ and F″)^27^. Samples were then subjected to SDS–PAGE and autoradiography (top panels). Bottom panels: the intensities of bands corresponding to the full-length form of Tom40 and Porin, the fragment F′ of Tob55 or the mature form of pSu9-DHFR from three independent experiments were quantified and the mean intensity from the import without any peptide was set to 100%. Error bars represent standard deviation. The precursor and mature forms of pSu9-DHFR are indicated by p and m, respectively. (**f**) The indicated radiolabelled proteins were incubated with isolated mitochondria in the presence of various amounts of the cyclic peptide. The import reactions were analyzed as in parts (**b**) and (**d**). AAC, ADP-ATP carrier.

In the present study we investigated whether a β-hairpin fold can serve as a targeting signal to direct β-barrel proteins from the cytosol to the mitochondrial surface. To that goal, peptides derived from the human voltage-dependent anion channel 1 (hVDAC1) were chemically synthesized. hVDAC1 was chosen since its atomic structure was solved^23^,^24^, allowing realistic design of peptides corresponding to a β-hairpin motif or its fragment. Three peptides, which differ in their length and potential to form a β-hairpin motif, were used (Fig. 1a). The short linear peptide comprises only the last β-strand of hVDAC1 (a.a. N269 to L279) and is therefore not able to form a β-hairpin. The long linear peptide on the other hand corresponds to the last two β-strands and the loop between them (a.a. L257 to L279) and should be able to adopt a β-hairpin fold. We created a third peptide by extending the C terminus of the long linear peptide by two Pro residues and then this new C terminus was covalently linked to the N terminus, thus cyclizing the peptide (CYC). This cyclization increases the likelihood that the peptide will form a β-hairpin motif. To allow direct comparison, also the two linear peptides were extended by two Pro residues.

To analyze whether the cyclic peptide has indeed a more stable conformation, the long linear and the cyclic peptides were investigated by solution NMR spectroscopy. Although these measurements were not performed in a membrane-mimetic environment like those in which the atomic structure of VDAC was determined, they reflect the cytosolic milieu where the targeting signal should be recognized by the import receptors. Low spectral dispersion of the amide resonances as well as averaged ^3^J(HN,Hα) coupling constants point to an averaged, partially structured conformation of both peptides (Supplementary Fig. 1a). As expected, differences in amide proton chemical shifts between the cyclic and the linear peptide are observed for residues at the termini close to the cyclization site (Supplementary Fig. 1b). However, also more distant residues in the C- and in particular in the N-terminal region between residues Leu3 and Gly9 showed marked chemical shift differences suggesting structural variations between the two peptides. In general, the cyclic peptide exhibits larger chemical shift dispersion for resonances in the two termini (Supplementary Fig. 1c). Taken together, the structural analysis shows that, as anticipated, the cyclic peptide is more structured than the linear one.

### Cyclic β-hairpin peptide hampers import of β-barrel proteins

We rationalized that a peptide resembling a targeting signal for mitochondrial β-barrel proteins should be able to hinder the *in organello* import of such proteins due to its ability to compete for crucial binding sites. To test this assumption, we added increasing amounts of the three peptides to an *in organello* import reaction containing isolated yeast mitochondria and radiolabelled β-barrel proteins like Tom40, Tob55/Sam50 and Porin. The addition of both linear peptides did not significantly affect the import of the radiolabelled proteins. In contrast, the cyclic peptide demonstrated a dose-dependent capacity to dramatically inhibit the import of all three tested β-barrel proteins (Fig. 1b–d). Since both Tom40 and Porin belong to the VDAC-like branch of mitochondrial β-barrel proteins and Tob55 to the Omp85-like protein family, the observed inhibition capacity suggests that a β-hairpin motif is a general targeting signal.

To assure that the peptides do not eliminate the general import capacity of the isolated organelles, we imported the matrix-destined protein pSu9-DHFR in the presence of the peptides and observed only a slight inhibition with the highest amount of the cyclic peptide (Fig. 1e). This minor inhibition is probably due to the interaction of the peptide with the import receptor Tom20 that recognizes also matrix-destined preproteins (see below). β-Barrel proteins and multispan inner membrane proteins are stabilized during their import by small Tim chaperones in the IMS^25^,^26^,^27^. Thus, a possibility that we wanted to exclude was that the import inhibition by the cyclic peptide is due to its ability to rupture the OM and thus to cause release of the small Tim components. However, whereas the import of the β-barrel proteins was strongly affected by the cyclic peptide only a minimal effect on the import of the inner membrane protein ADP–ATP carrier was observed (Fig. 1f). This finding suggests that the small Tims are maintained by the organelles in the presence of the cyclic peptide. Hence, the cyclic peptide specifically inhibits the import of β-barrel proteins.

### β-Hairpin motif is sufficient for mitochondrial targeting

**Figure 2:**
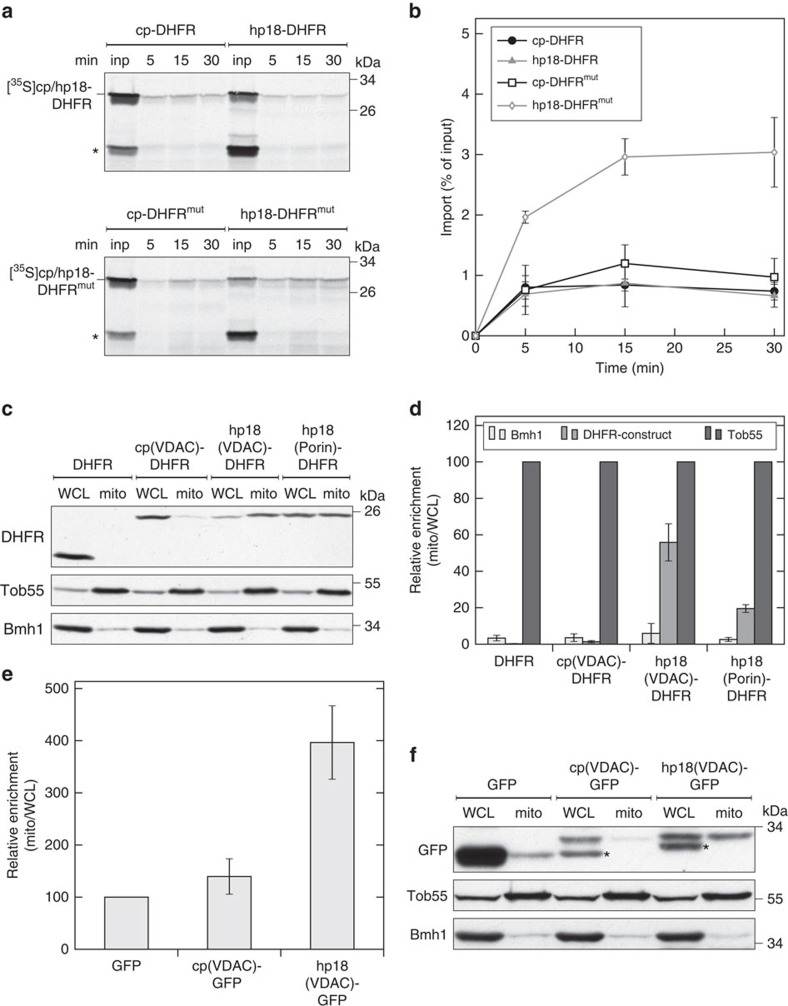
β-Hairpin peptides can target passenger proteins to mitochondria. (**a**) The specified radiolabelled fusion proteins were imported into isolated mitochondria for the indicated time periods. An asterisk marks a translation product generated from an internal ATG codon corresponding to DHFR or DHFR^mut^. inp, 5% lysate input. (**b**) The intensity of the bands from three independent experiments as in (**a**) was quantified and plotted against the time of the import reaction. Error bars represent s.d. (**c**) Crude mitochondria were isolated from yeast cells expressing DHFR alone or the indicated fusion proteins. Samples from the whole-cell lysate (WCL) and the crude mitochondria (mito) were analyzed by SDS–PAGE and immunodecoration with antibodies against the indicated proteins. Tob55, mitochondrial β-barrel protein; Bmh1, cytosolic protein. (**d**) The intensity of the bands from three independent experiments as in (**c**) was quantified and the mitochondrial enrichment was calculated by dividing the signal in the mitochondrial fraction by the one in the whole-cell lysate. The mitochondrial enrichment of Tob55 was set to 100. The results represent mean±s.d. from three independent experiments. (**e**) Samples from the whole-cell lysate and crude mitochondrial fractions of yeast cells expressing the indicated proteins were analyzed for their GFP fluorescence. The mitochondrial enrichment of the GFP signal was calculated and the one of GFP-expressing cells was set to 100. The results represent mean±s.d. from three independent experiments. (**f**) The samples from the experiments in (**e**) were analyzed by SDS–PAGE and immunodecoration with antibodies against the indicated proteins. An asterisk marks a shorter form of the fusion proteins resulting probably from degradation.

The aforementioned results suggest that a β-hairpin peptide can compete the targeting of a β-barrel protein to mitochondria. We next asked whether such a motif can mediate also a translocation across the OM. To that goal, fusion proteins containing the most C-terminal β-hairpin segment (hp18) of hVDAC1 upstream of the soluble dihydrofolate reductase (DHFR) domain were constructed. As a control, the native DHFR domain fused to a control peptide (cp), derived from the hVDAC1 N terminus, which forms a helical structure^23^,^24^, was employed. Next, radiolabelled hp18-DHFR or cp-DHFR were incubated with isolated mitochondria and the samples were then treated with proteinase K (PK) to remove non-imported material. In both cases only marginal amounts of the fusion proteins were protease protected (Fig. 2a,b). We reasoned that the tightly folded DHFR domain prevents full import into the interior of the organelle and thus replaced the native DHFR by a variant that cannot fold properly (DHFR^mut^)^28^. A fusion protein of DHFR^mut^ with a β-hairpin motif could indeed be imported into a protease-protected location whereas the control peptide did not mediate this effect (Fig. 2a,b). Our findings demonstrate that a β-hairpin motif can function *in organello* as a mitochondrial targeting signal.

Next, we asked whether peptides, which represent the last β-hairpin (hp18) of either hVDAC1 or yeast Porin, are able to mediate also *in vivo* mitochondrial targeting of a passenger domain. To that end, the fusion proteins described above and native DHFR moiety without any additive peptide were expressed in yeast cells and the capacity of the fusion proteins to associate with mitochondria was tested by analyzing proteins found with the fraction of crude mitochondria upon subcellular fractionation. As expected, DHFR alone and the cytosolic marker protein Bmh1 were detected in the whole-cell lysate but hardly in the mitochondrial fraction. Similarly, only negligible amounts of the construct with the control peptide associated with the organelles. In contrast, both fusion proteins containing the β-hairpin motif were enriched in the mitochondrial fractions (Fig. 2c,d). Of note, fusion of the β-hairpin peptides C-terminally to DHFR resulted in a lower mitochondrial targeting capacity (Supplementary Fig. 2). It might be that the β-hairpin peptides are not properly exposed in these latter fusion proteins.

To substantiate the *in vivo* targeting capacity, we constructed fusion proteins of hp18(VDAC) or the N-terminal control peptide with green fluorescent protein (GFP). Fluorescence microscopy analysis of cells expressing these proteins demonstrated strong GFP background in the cytosol that prevented observation of clear mitochondrial structures. To overcome this problem we isolated crude mitochondria from lysates of these cells and analyzed the samples by measuring their GFP fluorescence with a fluorimeter or by analyzing the samples with immunodecoration with an anti-GFP antibody. No enrichment of the GFP fluorescence signal on mitochondria was observed for cells with GFP alone or fused to the control peptide. In contrast, about threefold enrichment of the fluorescent signal was measured for mitochondria from cells containing the hp18(VDAC)-GFP construct (Fig. 2e). Accordingly, only the GFP fusion construct containing hp18(VDAC) was detected in the mitochondrial fraction by western blotting (Fig. 2f). Collectively, we conclude that such a β-hairpin motif is sufficient for mitochondrial targeting *in vivo*.

### β-Hairpin redirects chloroplast OM proteins to mitochondria

**Figure 3:**
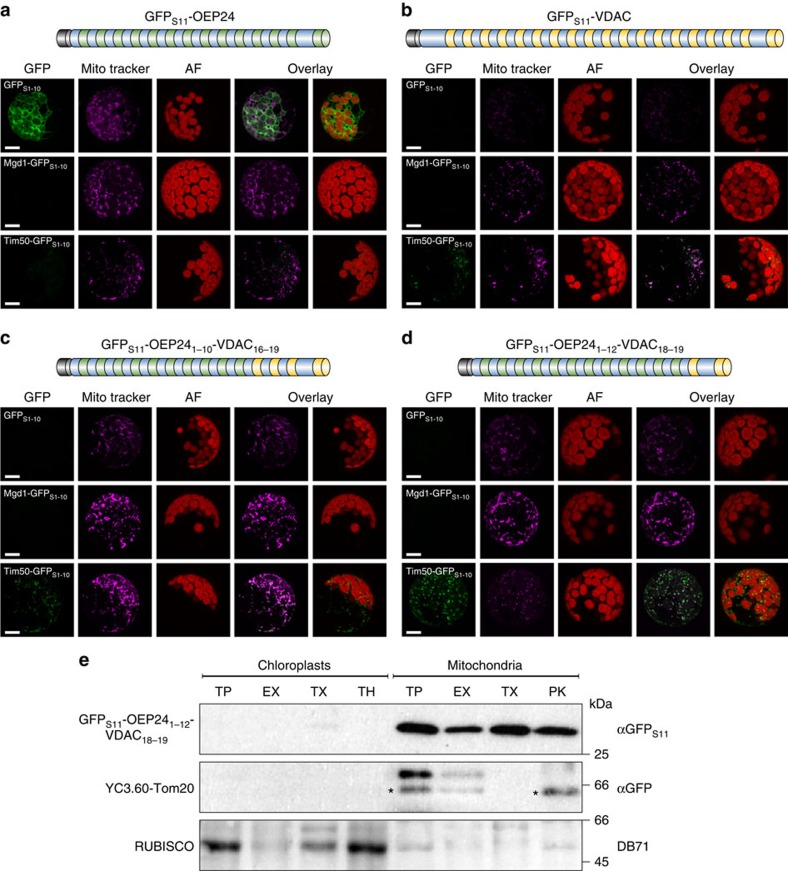
The last β-hairpin of atVDAC1 directs psOEP24 to mitochondria. (**a**–**d**) The specified fusion proteins and the indicated reporter constructs were co-transformed into *A. thaliana* protoplasts. GFP, MitoTracker and chlorophyll autofluorescence (AF) signal as well as the overlays of GFP with either of the latter two for a representative image from at least three independent experiments is shown. Schemes of psOEP24 and constructs generated are shown. Yellow/green sections indicate transmembrane β-strands of atVDAC1 and psOEP24, respectively, and the black section represents the GFP_S11_ strand. Scale bar, 10 μm. (**e**) Chloroplasts and mitochondria fractions were gained from protoplasts co-transfected with GFP_S11_-OEP24_1–12_-VDAC_18–19_ and YC3.60-Tom20. Samples of the organelles were analyzed directly (total protein, TP) or they were subjected to carbonate extraction and the pellets were loaded (EX). Other samples were solubilized by Triton X-100 (TX), or treated with thermolysin (TH) or PK. Next, the organelles were analyzed by SDS–PAGE and immunodecoration with the indicated antibodies. DB71 stain of RUBISCO is shown as loading control. YC3.60 is fused to the N terminus of Tom20 and the cytosolic domain of Tom20 is removed by PK. Bands resulting from cross-reactivity of the GFP antibody are marked with an asterisk.

Our aforementioned experiments were performed in yeast cells, and we next wanted to investigate whether the targeting capacity of the β-hairpin motif is conserved in higher eukaryotes. Plant cells provide an optimal experimental system, since in contrast to other eukaryotes, they contain two organelles with membrane-embedded β-barrel proteins, namely, chloroplasts and mitochondria. Thus, mitochondrial β-barrel proteins in such cells must avoid mistargeting to chloroplasts. To study the importance of the C-terminal β-hairpin for mitochondrial targeting, we generated hybrid constructs where the last two or four transmembrane β-strands of the pea chloroplast OM β-barrel protein psOEP24 were replaced by the corresponding C-terminal transmembrane β-strands of the *Arabidopsis* mitochondrial OM β-barrel protein atVDAC1 (Fig. 3). The positions of the β-strands of atVDAC1 were assigned based on an alignment to the mouse VDAC1 sequence. The positions of the β-strands of psOEP24 were predicted as described in the Methods section. To study the location of the hybrid β-barrel proteins *in vivo*, we employed the established self-assembly-GFP assay, where the first 10 β-strands of GFP (GFP_S1–10_) are fused to one protein, whereas the complementing 11th β-strand (GFP_S11_) is attached to another protein. A GFP signal can be observed only if the two fusion proteins are located in the same cellular compartment^29^,^30^.

We first confirmed that the N terminus of psOEP24 is exposed to the cytosol using a GFP_S11_-OEP24 construct. GFP fluorescence could be established only when the latter construct was co-expressed with cytosolic GFP_S1–10_. In contrast, no fluorescence signal was obtained upon co-expression with either Mgd1-GFP_S1–10_, which is targeted to the IMS of chloroplasts, or with Tim50-GFP_S1–10_ that is targeted to the mitochondrial IMS (Fig. 3a). These observations confirm the previously observed exclusively plastidic localization of psOEP24 (ref. 31). Next, we analyzed the localization of the mitochondrial atVDAC1. GFP_S11_-VDAC1 was co-expressed with each of the aforementioned three GFP_S1–10_-containing reporter constructs and GFP fluorescence was observed only upon co-expression with mitochondrial Tim50-GFP_S1–10_. This signal co-localized with MitoTracker-stained mitochondria (Fig. 3b). Hence, the N terminus of atVDAC1 is localized in the mitochondrial IMS.

Next, we analyzed the intracellular location of hybrid proteins composed of the N-terminal portion of psOEP24 and C-terminal β-hairpins of atVDAC1. Interestingly, constructs where the last two or four β-strands of psOEP24 were replaced by β-strands from the mitochondrial atVDAC1 (OEP24_1–12_-VDAC1_18–19_ or OEP24_1–10_-VDAC1_16–19_, respectively, subscript numbers reflect the number of the β-strand in the corresponding protein), assemble fluorescent GFP only upon co-expression with the mitochondrial IMS marker and this staining co-localizes with the MitoTracker signal (Fig. 3c,d). These observations indicate that these hybrid proteins are not targeted to chloroplasts but rather to mitochondria. Of note, the N terminus of these fusion proteins apparently faces the mitochondrial IMS suggesting that although the C terminus of the mitochondrial atVDAC1 can mediate mitochondrial targeting, these fusion proteins are not inserted into the mitochondrial OM in a psOEP24-like topology.

To support our findings with an additional example, we fused GFP_S11_ to a hybrid protein composed of β-strands 1–16 of the chloroplast β-barrel protein psOEP37 and the last β-hairpin of atVDAC1 (GFP_S11_-OEP37_1–16_-VDAC1_18–19_). Similar to our observation with psOEP24, also this hybrid protein was targeted to mitochondria (Supplementary Fig. 3). Thus, it appears that a mitochondrial β-hairpin has a general capacity to target either soluble proteins (DHFR and GFP) or plastidic proteins (OEP24 and OEP37) to mitochondria.

To confirm the mitochondrial localization of the fusion protein GFP_S11_-OEP24_1–12_-VDAC1_18–19_ and to evaluate whether it is indeed inserted into the mitochondrial OM, protoplasts from *Arabidopsis* cells expressing it were fractionated into samples enriched in either chloroplasts or mitochondria. To control for the efficiency of the organelle enrichments, protoplasts co-expressed the mitochondrial OM protein YC3.60-Tom20 (ref. 31). We detected the hybrid β-barrel protein in mitochondria but not in chloroplasts (Fig. 3e). A portion of the hybrid was partially resistant to either carbonate extraction or externally added PK. In contrast, the cytosolic domain of the control protein YC3.60-Tom20 was removed by PK treatment. However, when mitochondria were solubilized by Triton X-100 (TX), we could detect also a substantial portion of the hybrid protein in the pellet fraction suggesting that these molecules were aggregated (Fig. 3e). Hence, it seems that the hybrid protein can be perfectly targeted to mitochondria but cannot be completely integrated into the membrane and thus remains partially aggregated. Collectively, these findings demonstrate that the last β-hairpin of atVDAC1 is sufficient to target a chloroplast β-barrel protein to mitochondria.

We next wondered whether the capacity to assure mitochondrial targeting is limited to the last β-hairpin from plant atVDAC1 or can be extended to a similar β-hairpin from its yeast homologue scPorin. To that goal we created the hybrid proteins GFP_S11_-Porin and a construct where the last β-hairpin of psOEP24 is replaced by the last β-hairpin of yeast Porin (GFP_S11_-OEP24_1–12_-Porin_18–19_) and expressed them in protoplasts. GFP_S11_-Porin partially aggregates in the cytoplasm, but its majority is localized to mitochondria as judged from the fluorescence signal upon co-expression with Tim50-GFP_S1–10_ and the overlay of the GFP staining with the MitoTracker signal (Supplementary Fig. 4a). As seen for GFP_S11_-Porin, co-expression of GFP_S11_-OEP24_1–12_-Porin_18–19_ with GFP_S1–10_ shows that the fusion protein partially aggregates in the cytoplasm. However, co-expression of this hybrid protein with Tim50-GFP_S1–10_, but not with Mgd1-GFP_S1–10_ yielded efficient GFP assembly and co-localization of the GFP fluorescence with mitochondria (Supplementary Fig. 4b). These observations suggest that this hybrid protein can be targeted to mitochondria, thereby verifying that the last β-hairpin of yeast Porin is sufficient for mitochondrial targeting. This conclusion is supported by fractionation of the transformed cells and analysis of the mitochondrial and chloroplasts fractions. GFP_S11_-OEP24_1–12_-Porin_18–19_ was exclusively localized to mitochondria and a major portion of the protein was resistant to carbonate extraction and PK treatment. However, a substantial fraction of the fusion protein is also aggregated as it cannot be solubilized by Triton X-100 treatment (Supplementary Fig. 4c).

To analyze the membrane integration and the location of the N-termini of the various hybrid proteins in additional ways, we first tested the protection of the various constructs to protease treatment of whole protoplast lysate. The N terminus of GFP_S11_-OEP24, which is targeted to chloroplasts, is exposed to the cytosol and thus was accessible to the protease. In contrast, GFP_S11_-tagged versions of either atVDAC1 or scPorin and the hybrid proteins of their last β-hairpin with psOEP24 were resistant to such treatment. This resistance was dramatically reduced upon solubilization of organelles with detergent suggesting that the majority of the protection was not solely caused by aggregation (Supplementary Fig. 5a). Thus, it appears that the hybrid proteins are not only targeted to mitochondria but are also able to reach a protease-protected location.

Next, we wanted to confirm, by using an alternative GFP_S1–10_ mitochondrial reporter protein, that the N terminus of the various hybrid proteins is indeed accessible at the mitochondrial IMS. To that goal we constructed a protein that includes the N-terminal region of Tim21-like1 (Tim21(N)), which harbours the mitochondrial import signal and the first transmembrane domain of the protein, and fused it to GFP_S1–10_. In line with the aforementioned results, co-expression of this reporter with GFP_S11_-tagged atVDAC1 or scPorin and with their hybrid proteins, but not with GFP_S11_-OEP24, gave a mitochondrial staining that co-localizes with the MitoTracker signal (Supplementary Fig. 5b). These observations verify that the last β-hairpin of either atVDAC1 or scPorin is sufficient to target a chloroplast β-barrel protein to mitochondria.

### The β-signal is not sufficient for mitochondrial targeting

**Figure 4:**
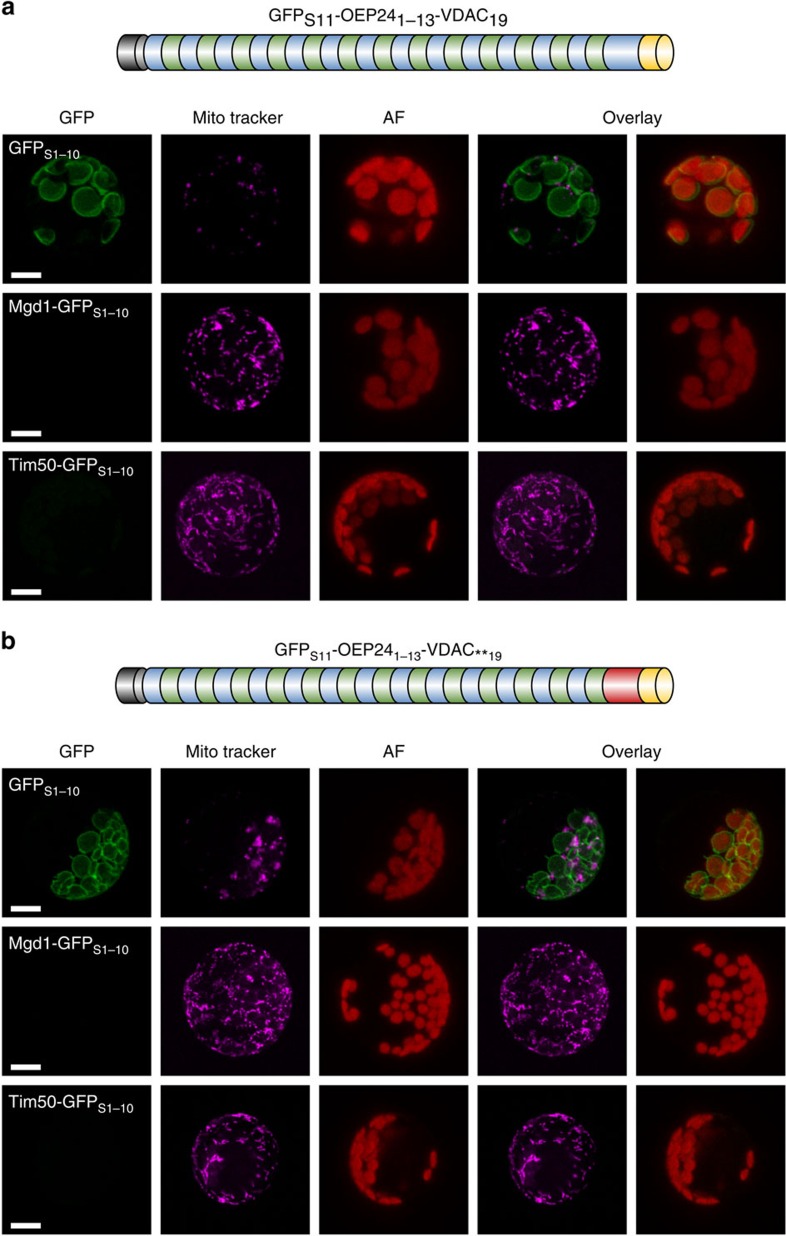
The ultimate β-strand of atVDAC1 does not direct psOEP24 to mitochondria. (**a**, **b**) The last β-strand (GFP_S11_-OEP24_1–13_-VDAC_19_) (**a**) or the last β-strand and the last loop (GFP_S11_-OEP24_1–13_-VDAC_**19_) (**b**) of psOEP24 were replaced by the corresponding regions of atVDAC1 and the resulting fusion proteins were co-transformed with the indicated reporter constructs into *A. thaliana* protoplasts. Signals and schemes are as in Fig. 3a. Scale bar, 10 μm.

We next asked whether even a single β-strand from the C terminus of atVDAC1 is sufficient to assure mitochondrial targeting. This last strand contains the β-signal, which was proposed to be important for sorting within mitochondria^14^. To address this question we initially replaced either the last β-strand (GFP_S11_-OEP24_1–13_-VDAC_19_) or the last β-strand together with the last loop of psOEP24 (GFP_S11_-OEP24_1–13_-VDAC_**19_) by the corresponding regions of atVDAC1. Co-expression of these proteins in protoplasts with the markers for the organellar IMS did not result in an observable signal. In contrast, co-expression of both hybrid proteins with the cytosolic GFP_S1–10_ yielded GFP fluorescence surrounding chloroplasts (Fig. 4). Thus, the last β-strand is not sufficient to target hybrid proteins to mitochondria.

### The last β-hairpin is necessary for mitochondrial targeting

**Figure 5:**
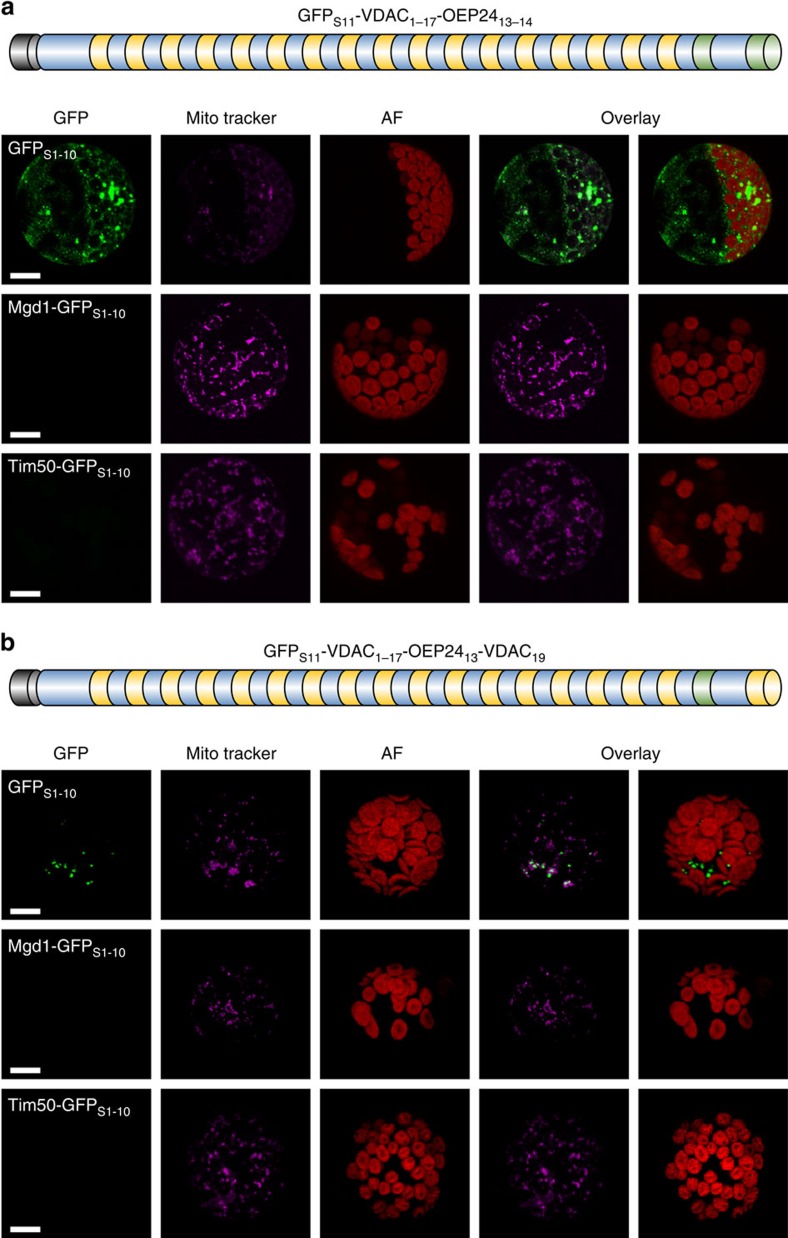
The last β-hairpin of atVDAC1 is important for mitochondrial targeting. (**a**,**b**) GFP_S11_-VDAC_1–17_-OEP24_13–14_ (**a**) or GFP_S11_-VDAC_1–17_-OEP24_13_-VDAC_19_ (**b**) and the indicated reporter constructs were co-transformed into *A. thaliana* protoplasts. Signals and schemes are as in Fig. 3a. Scale bar, 10 μm.

Our aforementioned results clearly demonstrate that the last β-hairpin is sufficient by itself for mitochondrial targeting of non-mitochondrial proteins. We next asked whether this segment is also necessary to target native mitochondrial β-barrel proteins. To that end, we replaced either the last two β-strands of atVDAC1 by the last two β-strands of psOEP24 resulting in the construct GFP_S11_-VDAC_1–17_-OEP24_13–14_ (subscript numbers reflect the number of the β-strand in the corresponding protein) or we replaced only the penultimate β-strand of atVDAC1 by the corresponding penultimate strand of psOEP24 (GFP_S11_-VDAC_1–17_-OEP24_13_-VDAC_19_). Co-expression of these fusion proteins with the various reporter constructs revealed GFP fluorescence exclusively with the cytosolic marker GFP_S1–10_. Moreover, the GFP stain did not co-localize with mitochondria (Fig. 5). Thus, the complete last β-hairpin is required for mitochondrial targeting.

We next wondered if the last β-hairpin is also necessary for the targeting and assembly of mammalian VDAC1. To that goal we used the clear growth phenotype at elevated temperatures of yeast cells deleted for *POR1* (refs 30, 32). hVDAC1 or its variants where the first (VDAC1Δhp1, lacking β-strands 1 and 2) or last (VDAC1Δhp18, lacking β-strands 18 and 19) β-hairpin is missing were expressed in wild type or *por1*Δ yeast cells and the growth behaviour of the transformed cells was monitored. None of the three constructs had any effect on wild type cells but only the full-length construct and the one lacking the first β-hairpin complemented the absence of yeast Porin. In contrast, the truncated variant without the last β-hairpin demonstrated only a very partial complementation capacity and could not support any growth on a non-fermentable carbon source (YPG) where fully functional mitochondria are required for viability (Supplementary Fig. 6a). Accordingly, VDAC1Δhp18 was detected in much lower levels than the native protein and VDAC1Δhp1 (Supplementary Fig. 6b), suggesting lower stability and higher turnover rate of the former variant. Our findings thus substantiate the importance of the last β-hairpin in the targeting of human mitochondrial β-barrel proteins.

### Hydrophobicity of the β-hairpin is crucial for targeting

**Figure 6:**
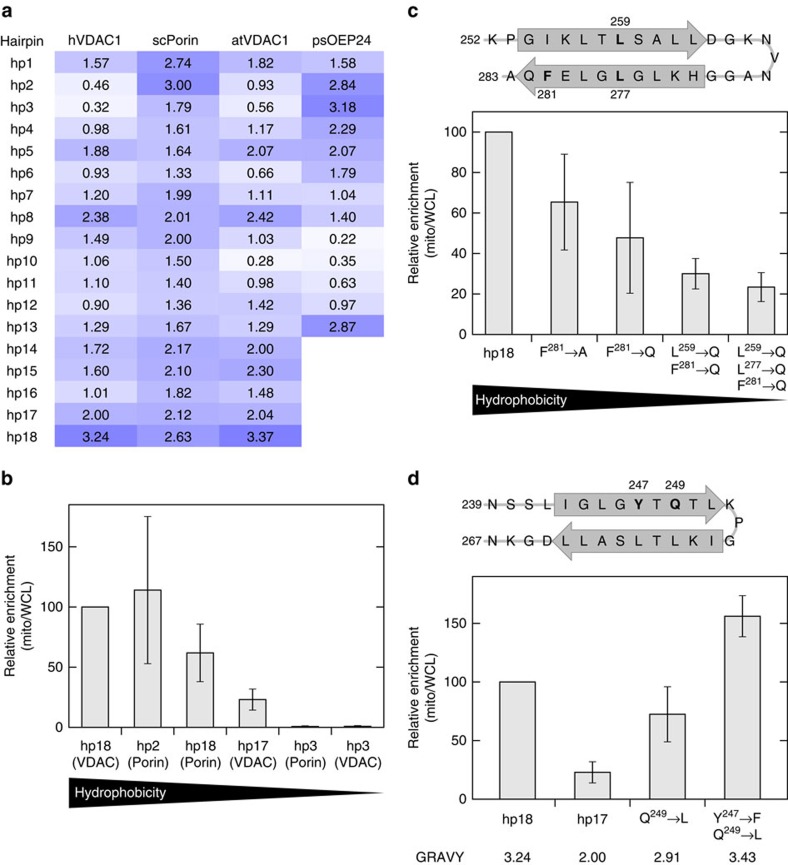
The hydrophobicity of the β-hairpin motif is important for its mitochondrial targeting efficiency. (**a**) The average hydrophobicity of the hydrophobic face of the β-hairpins of various β-barrel proteins was calculated by summing up the hydropathy values of the residues facing the lipid environment from both β-strands that form each β-hairpin and dividing it by the number of the residues. (**b**) Yeast cells expressing the indicated β-hairpin peptides fused to DHFR were analyzed as in Fig. 2c,d. The mitochondrial enrichment of hp18(VDAC)-DHFR was set to 100. The results represent mean±s.d. from three independent experiments. (**c**) Top panel: scheme of hp18(VDAC) used in these experiments. Grey arrows indicate the residues that form a β-strand in full-length hVDAC1. Residue numbers correspond to the numbering of full-length hVDAC1. Amino acids that were mutated are shown in bold font. Bottom panel: yeast cells expressing hp18(VDAC) peptide or its variants with the indicated mutations fused to DHFR were analyzed as in Fig. 2c,d. The mitochondrial enrichment of the control hp18-DHFR was set to 100. The results represent mean±s.d. from three independent experiments. (**d**) Top panel: scheme of hp17(VDAC) depicted as in (c). Bottom panel: yeast cells expressing DHFR fused to hp18(VDAC) peptide, hp17(VDAC) peptide or variants of hp17(VDAC) with the indicated mutations were analyzed as in Fig. 2c,d. The mitochondrial enrichment of the hp18-DHFR protein was set to 100. The results represent mean±s.d. from three independent experiments. GRAVY, grand average hydrophobicity of the hydrophobic face of the hairpin.

Since many proteins, including the widely used GFP and its variants, contain β-hairpin motifs but only *bona fide* mitochondrial β-barrel proteins assemble into the mitochondrial OM, we wondered which requirements a β-hairpin motif should fulfil in order to serve as a mitochondrial targeting signal. The membrane-embedded β-strands of β-barrel proteins have amphipathic structures where those amino acids facing the lumen of the barrel are rather hydrophilic, whereas those that face the lipid core are more hydrophobic. Inspection of the amino acids that build the hydrophobic face of hp18 of yeast Porin and hVDAC1 and of hp2 of yeast Porin (containing β-strands 18/19 or 2/3, respectively) suggests that these residues are on average more hydrophobic than the lipid-facing amino acids in other β-hairpins (Fig. 6a). To study the significance of this difference, we fused the passenger domain DHFR to peptides resembling β-hairpins with various hydrophobicities (hairpins 3, 17 and 18 of hVDAC1 and 2, 3 and 18 of Porin). When we then compared the mitochondrial association capacity of the resulting fusion proteins, we observed a clear correlation between the hydrophobicity of the lipid-facing residues of the peptides and the ability of the corresponding peptide to target the passenger domain to mitochondria (Fig. 6b).

Assuming that hydrophobicity is important for the targeting ability of a β-hairpin motif, mutations at the lipid-facing side that replace amino acids by less hydrophobic ones should decrease the targeting capacity of the corresponding β-hairpin motif. To test this prediction, we created several DHFR-containing hybrids where one, two or three of the hydrophobic residues in hp18 of hVDAC1 were replaced by the polar residue Gln (Fig. 6c). When we monitored the mitochondrial association of these hybrids, we observed a clear correlation between the number of substituted amino acids and a reduction in the mitochondrial targeting capacity. The targeting capability of constructs with two or three inserted Gln residues was reduced by about 70 or 80%, respectively (Fig. 6c).

Along this line, we anticipated that increasing the hydrophobicity of the lipid-facing side should enhance the mitochondrial targeting capacity of the resulting β-hairpin. Indeed replacing in hp17 of hVDAC1 (containing β-strands 17/18) the polar residue Gln249 by Leu resulted in a clear enhancement in the mitochondrial association of the obtained construct. Moreover, adding to the above mutation also the Tyr247 to Phe replacement resulted in a further considerable augmentation of the mitochondrial binding of the resulting hybrid protein (Fig. 6d).

To substantiate this point also in plant cells, we constructed a hybrid protein where the fifth and sixth β-strands of psOEP24 were replaced by the corresponding β-strands of atVDAC1 (GFP_S11_-OEP24_1–4_-VDAC_5–6_-OEP24_7–14_). The average hydrophobicity of these β-strands of atVDAC1 is lower than that of strands 18 and 19 of the protein (2.1 as compared to 3.4, respectively). When this hybrid protein was co-expressed in protoplasts together with the various marker proteins it aggregated in the cytosol and was not targeted to mitochondria (Supplementary Fig. 7). Collectively, these findings demonstrate that elevated hydrophobicity of the lipid-facing side of a β-hairpin is crucial for its function as a mitochondrial targeting signal.

### The import receptor Tom20 recognizes the β-hairpin signal

**Figure 7:**
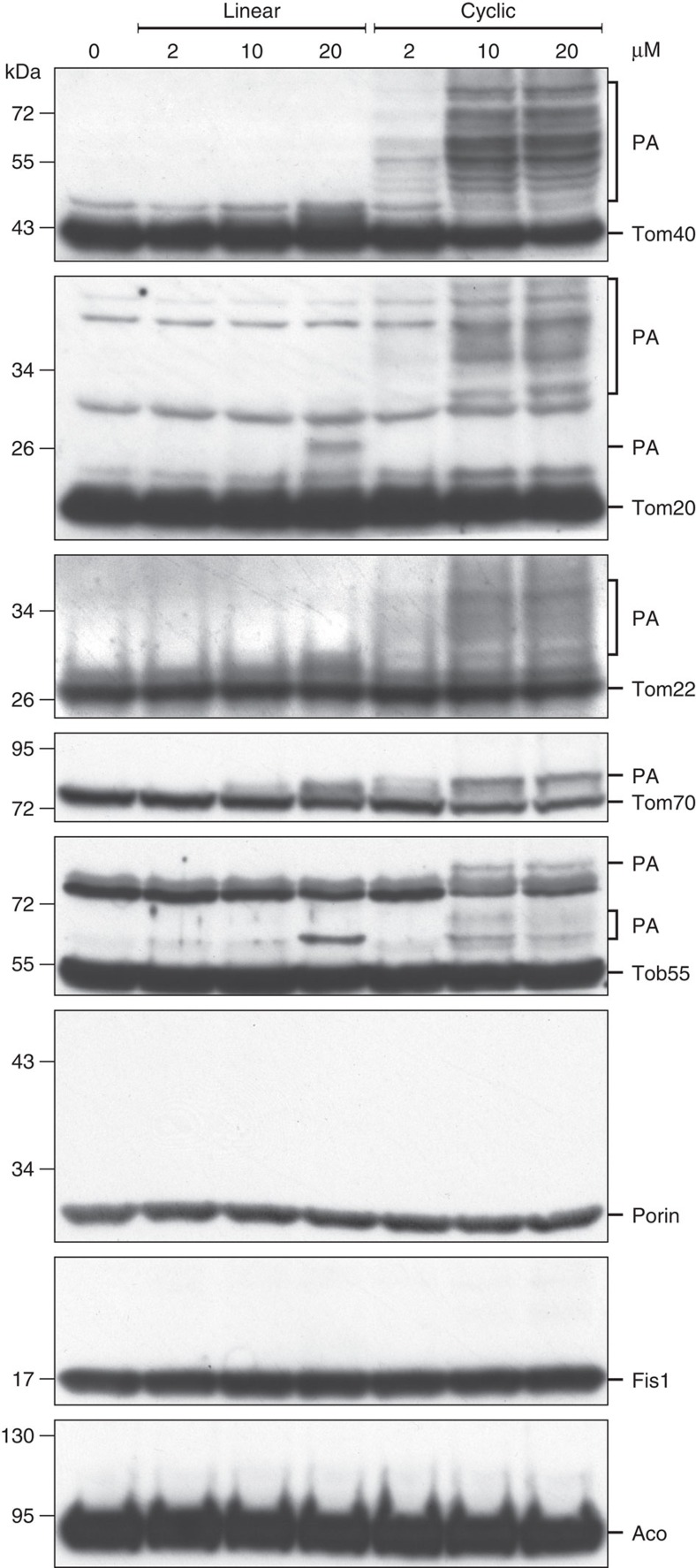
The cyclic β-hairpin peptide can be cross-linked *in organello* to components of the TOM complex. Isolated mitochondria were incubated with a Bpa-containing linear or cyclic peptide at the indicated concentrations. After ultravoilet-induced cross-linking the samples were analyzed by SDS–PAGE and immunodecoration with antibodies against the indicated proteins. PA, photo-adducts.

The establishment of a β-hairpin as a mitochondrial targeting signal raised the question, which import elements can recognize it. To address this question, we synthesized the β-hairpin peptide in its linear or cyclic forms and replaced Leu263 by the photo-reactive benzoyl-phenylalanine (Bpa) moiety. Ultraviolet-activation of Bpa results in generation of covalent bonds between the photo-peptide and proteins in its vicinity. Next, we mixed various amounts of the photo-peptides with isolated mitochondria and performed photo-crosslinking. Subsequent western blot analysis demonstrated crosslinking adducts of the cyclic β-hairpin peptide with import components like Tom40, Tom22 and Tom70 (Fig. 7). As anticipated, formation of photo-adducts was also observed with the import receptor Tom20 that was suggested by several studies to be the major mitochondrial import receptor for β-barrel proteins^33^,^34^,^35^,^36^.

Several cross-linking adducts of the peptide and the various TOM components can be detected. We suppose that this variability can result from a variable number of peptides bound to one molecule of protein and from different crosslinking sites on the protein, which in turn can cause different migration behaviour in the SDS–PAGE. Of note, weak cross-linking adducts were also observed with Tob55 suggesting that a small portion of the peptide molecules was translocated across the OM and associated already with the TOB complex. The specificity of the cross-linking adducts is demonstrated by their absence when no peptide or the linear peptide were used and the lack of photo-adducts with unrelated mitochondrial proteins like Fis1, Porin or aconitase. Thus, the crosslinking assay demonstrates that the β-hairpin motif interacts with the TOM complex.

**Figure 8:**
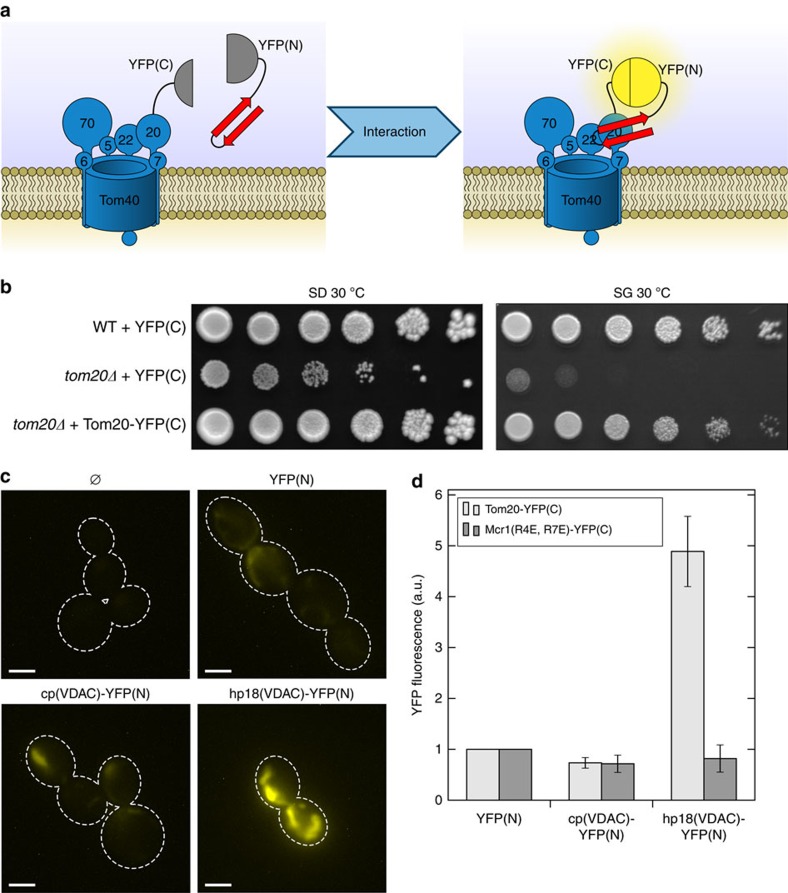
A β-hairpin peptide interacts with the import receptor Tom20 *in vivo*. (**a**) Schematic representation of the assay. Tom20 was fused with the C-terminal part of YFP (YFP(C)) whereas the N-terminal part of YFP (YFP(N)) was fused to the β-hairpin peptide (red). YFP fluorescence can be observed only upon interaction of the two fusion proteins. (**b**) The growth of WT and *tom20Δ* yeast cells expressing either YFP(C) or the fusion protein Tom20-YFP(C) was analyzed by drop-dilution assay. (**c**) Yeast cells expressing Tom20-YFP(C) alone (Ø), or co-expressing Tom20-YFP(C) with YFP(N), the control peptide (cp(VDAC)) fused to YFP(N) or hp18(VDAC) fused to YFP(N) were subjected to fluorescence microscopy. Representative images are shown. Scale bar, 5 μm. (**d**) Crude mitochondria were isolated from cells co-expressing the indicated YFP(N) fusion proteins and either Tom20-YFP(C) or Mcr1(R4E, R7E)-YFP(C). The YFP fluorescence of the crude mitochondrial fractions was analyzed and the signal of cells expressing YFP(N) without any additive was set to 1. Data represents mean±s.d. of three independent experiments.

To check *in vivo* the potential interaction of Tom20 with the β-hairpin motif, we employed a bimolecular fluorescence complementation assay (Fig. 8a). Either hp18 or the cp of hVDAC1 were fused to the N-terminal portion of YFP, whereas Tom20 was fused at its C terminus to the C-terminal part of YFP. We verified the functionality of Tom20-YFP(C) by demonstrating its capacity to complement the growth phenotype of cells lacking Tom20 (Fig. 8b). Furthermore, similarly to native Tom20, Tom20-YFP(C) is exposed to the cytosol as suggested by its availability to external protease and it is embedded in the OM as it cannot be extracted by alkaline solution (Supplementary Fig. 8). Thus, Tom20-YFP(C) acquires native-like topology and is fully functional.

Next, various combinations of pairs of fusion proteins were expressed in yeast cells and the cells were analyzed by fluorescence microscopy. As expected, cells harbouring Tom20-YFP(C) alone or in the presence of cytosolically-expressed YFP(N) demonstrated only basal fluorescence signal. Similarly marginal was the signal when Tom20-YFP(C) was co-expressed with the hVDAC1 N-terminal control peptide fused to YFP(N). In sharp contrast, a strong fluorescence staining of mitochondrial structures was observed in cells co-expressing Tom20-YFP(C) and hp18(VDAC)-YFP(N) (Fig. 8c). To control for the specificity of this interaction we fused YFP(C) to another OM protein with a similar topology like Tom20, namely Mcr1. This protein has two isoforms, a longer form that, similar to Tom20, is anchored to the mitochondrial OM via a single N-terminal segment and a shorter, processed one in the IMS^37^. As we previously observed that R4E and R7E mutations in Mcr1 cause an enhanced portion of the molecules in the OM^38^, we co-expressed Mcr1(R4E, R7E)-YFP(C) with different YFP(N)-containing fusion proteins. To obtain a reliable quantification of the fluorescence signal, crude mitochondria were isolated from the transformed cells and their fluorescence signal was measured with a fluorimeter. Our results clearly demonstrate that the co-expression of Tom20-YFP(C) and hp18(VDAC)-YFP(N) resulted in several fold stronger fluorescence signal in comparison to all other combinations (Fig. 8d). These findings strongly suggest that the β-hairpin targeting signal is either in close vicinity to Tom20 or physically interacting with the receptor protein.

**Figure 9:**
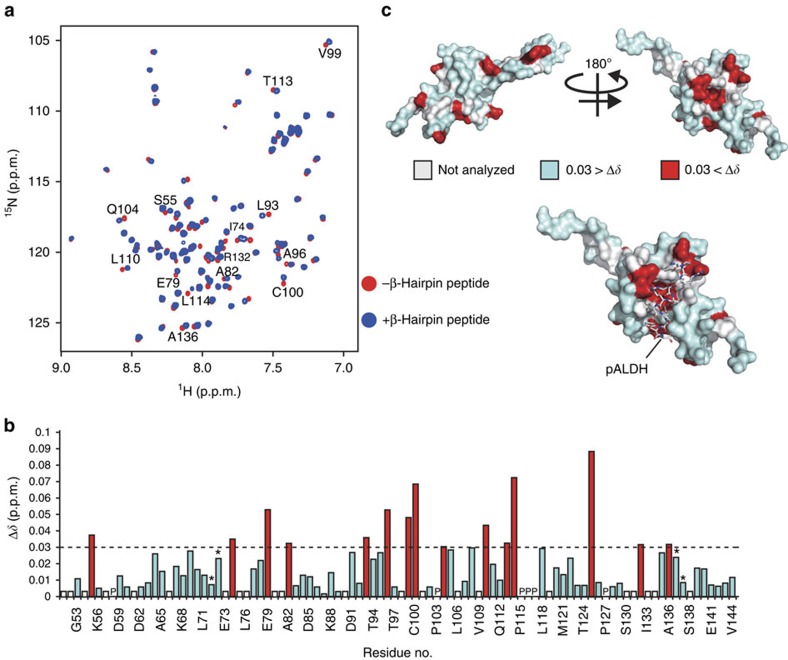
The β-hairpin peptide binds to the presequence binding region of Tom20. (**a**) [^1^H, ^15^N]-HSQC NMR spectra of [^15^N]-labelled rat dTom20 were recorded before (red spectrum) and after addition of two molar excess cyclic peptide (as in Fig. 1a) in DMSO (blue spectrum) or the same volumes of DMSO as control. (**b**) The chemical shift changes of each backbone amide of dTom20 in (**a**) are presented. Signals from several amino acids were not used for the analysis due to signal overlapping. The signals from some other residues were not assigned. These uncharacterized residues are shown in white. Signals showing chemical shift changes larger than 0.03  p.p.m. are shown in red and the others in pale blue. Asterisks, residues E72 and Q137 gave two signals each. (**c**) The residues whose signals show chemical shift changes larger or lower than 0.03  p.p.m. are shown in red and pale blue, respectively, on the surface model of rat dTom20 in a complex with the presequence peptide derived from rat aldehyde dehydrogenase (pALDH) (PDB ID 1OM2). The residues whose signals were not analyzed are shown in white. The structure of pALDH is shown as a stick model in the lower panel.

To directly probe the interactions of the cyclic β-hairpin peptide with the receptor Tom20 and to verify physical contact between the two, we monitored chemical-shift changes in the [^1^H, ^15^N]-HSQC NMR spectra of the uniformly [^15^N]-labelled cytosolic receptor domain of rat Tom20 (dTom20) upon addition of the non-labelled cyclic β-hairpin peptide. The peptide caused chemical-shift perturbations of a subset of the backbone amide signals of dTom20 (Fig. 9a,b). Interestingly, the dTom20 residues affected by the β-hairpin peptide are close to the presequence binding region of dTom20 (Fig. 9c; ref. 39), suggesting that the β-hairpin peptide shares with canonical presequences the same binding site on Tom20. These experiments demonstrate a direct interaction of the β-hairpin element with Tom20.

## Discussion

After their synthesis in the cytosol the vast majority of mitochondrial proteins are delivered to the mitochondrial surface with the help of an N-terminal, cleavable targeting signal, also known as presequence. In contrast, proteins residing in the OM of the organelle do not contain such a signal and their targeting is mediated by elements that are part of the mature protein. Efforts to identify linear sequences within β-barrel proteins that can fulfil this task have failed so far. Thus, it was assumed that the signals are contained in β-barrel-specific structural elements rather than in a conserved linear sequence. In this study we demonstrate that a β-hairpin motif with a highly hydrophobic face is the minimal structural element that can function as a mitochondrial targeting signal.

This motif fulfils all the requirements of a specific intracellular targeting signal. It is necessary for targeting as in its absence the remaining portion of the protein is not targeted properly to mitochondria. The signal is also sufficient by itself and can mediate mitochondrial location of soluble passenger domains. Furthermore, attachment of this signal to chloroplast β-barrel proteins redirects the latter to mitochondria. The β-hairpin motif is also the minimal signal and a single β-strand cannot fulfil this task. Finally, as expected from a targeting signal, the β-hairpin motif is recognized at the cytosolic side of the organelle by an import receptor (Tom20).

In most of the analyzed mitochondrial β-barrel proteins, the last β-hairpin at the C terminus has a high hydrophobicity and can serve as a targeting signal. This function can explain previous observations where mutations in the C-terminal region of the β-barrel proteins Porin and Tom40 hindered mitochondrial targeting of the mutated proteins, whereas mutations in the N-terminal segment did not have this effect^40^,^41^,^42^. The ultimate β-strand contains the previously identified β-signal, which is composed of several amino acids and is recognized by the TOB/SAM complex and serves as an intra-mitochondrial sorting signal^14^. Our finding that the minimal targeting signal is composed of two β-strands with the loop between them explains why the β-signal is neither sufficient nor absolutely required for the targeting from the cytosol to mitochondria. Point mutations in the β-signal affect interactions with the TOB/SAM complex but the mutated β-barrel proteins were still targeted from the cytosol to the organelle^14^. Along this line, our data show that a mutation that disrupts the β-signal (F281A) only moderately affects the targeting capacity of the resulting β-hairpin. Despite the special high targeting capacity of the last β-hairpin, it is probably not the only β-hairpin that can serve as a targeting signal in mitochondrial β-barrel proteins. In addition, in the fusion proteins used in the current study a signal fused N-terminally to a passenger domain could have a mitochondrial targeting capacity. Hence, the location of the signal within the substrate β-barrel protein appears not to be restricted to the most C-terminal β-hairpin. Accordingly, we found that fusion proteins containing the second β-hairpin of Porin are targeted to mitochondria in high efficiency. Furthermore, VDAC1 molecules deleted for the last β-hairpin were still detected on mitochondria (although only in minor amounts) suggesting that other β-hairpins can also fulfil the targeting task although with much lower potential. Hence, an avidity effect of several β-hairpins within the same protein can be anticipated.

The identification of a β-hairpin as a targeting signal can also explain how bacterial proteins that do not share any sequence homology with mitochondrial proteins can be targeted to mitochondria upon their expression in eukaryotic cells^16^,^17^,^18^,^19^. The β-hairpin motif is the most basic structural unit of all β-barrel proteins and thus is contained also by the bacterial ones^22^. Whereas in prokaryotes and non-plant eukaryotic cells β-barrel proteins are targeted only to one cellular membrane, the situation is more complicated in plant cells that harbour such proteins in two separate locations namely, mitochondria and chloroplasts. Hence, a mechanism that assures the specific targeting must have developed in such cells. We previously observed that plant plastidic β-barrel proteins show a clear discrimination between the two organelles only *in vivo*^30^. Our results demonstrate the existence of a specific signal that can target plant β-barrel proteins to mitochondria. However, it is unclear whether this signal and the yet to be discovered chloroplast signal are decoded by dedicated cytosolic guidance factors and/or specific chaperones. Obviously, mitochondrial proteins have to avoid recognition by chloroplasts targeting factors.

Irrespective of the cytosolic events, once the newly synthesized β-barrel protein reaches the surface of the organelle it is recognized by the TOM complex^4^,^13^,^43^. In line with these previous reports, the β-hairpin signal can be cross-linked to various components of the TOM complex suggesting that it can be in their vicinity. Previous studies suggested that the major receptor for these proteins is Tom20 that recognizes also presequence-containing mitochondrial proteins^33^,^35^,^36^,^44^. Accordingly, we observed by *in organello* and *in vitro* assays an interaction of the β-hairpin signal with Tom20. Remarkably, our NMR analysis indicated that the canonical presequence and the β-hairpin share the same binding region on Tom20. This similarity can be explained by the common amphiphilic structure of both elements where one face is rather polar and the other is hydrophobic. Our discovery regarding the shared binding site on Tom20 explains also the reduced *in vitro* import of presequence-containing mitochondrial protein upon addition of the cyclic β-hairpin peptide and previous observations where a peptide resembling a presequence could compete for the import of Porin^45^. In addition to Tom20, Tom22 and/or Tom70 were also suggested to be involved in processing of presequence-containing proteins^46^,^47^. Considering the structural similarity between the two signals and the observed cross-linking adducts of the β-hairpin peptide with the latter two proteins, one can speculate that Tom22 and/or Tom70 might also participate in the recognition of the β-hairpin element.

In summary, a dedicated β-hairpin motif is a newly-discovered genuine targeting signal that is necessary and sufficient for the targeting of mitochondrial β-barrel proteins to the organelle and for their recognition by import receptors.

## Methods

### Yeast strains and growth methods

Standard genetic techniques were used for growth and manipulation of yeast strains. The wild-type strain W303α was employed. The *tom20*Δ and *por1Δ* strains were described before (refs 18, 30, respectively).

### Drop-dilution assay

Cells were grown to logarithmic phase in appropriate liquid media, collected by centrifugation, and resuspended in water to an optical density at 600 nm of 2. Cell suspensions were serially diluted fivefold in water, and 5 μl aliquots of the cell suspensions were spotted on appropriate plates, which were then incubated at either 30 or 37 °C.

### Recombinant DNA techniques

The plasmids pGEM4-pSu9-DHFR, pGEM4-pSu9-DHFR^mut^ or pYX142-mtGFP were used as templates for the amplification of the DHFR or the green fluorescent protein genes by PCR. The amplification products were inserted into pGEM4 using KpnI and BamHI restriction sites. Fusion proteins containing segments of either hVDAC1 or yeast Porin were generated by PCR using as template the plasmid pGEM4-hVDAC1 or pGEM4-Por1, respectively. The sequences of all PCR primers used in this study are included in Supplementary Table 1. The DNA encoding these peptides was inserted into the previously generated pGEM4-DHFR, pGEM4-DHFR^mut^ or pGEM4-eGFP using EcoRI and KpnI restriction sites. For expression in yeast cells the DHFR or eGFP fusion constructs were sub-cloned into pYX142 using EcoRI and SalI restriction sites. The mutations within hp17(VDAC) and hp18(VDAC) were introduced via site-directed mutagenesis using the QuickChange Site-Directed Mutagenesis Kit (Stratagene) according to the manufacturer's instructions.

The YFP(C) constructs for the BIFC assays were amplified from pRS426-TPI-Tom20 and pGEM4-Mcr1 using standard PCR techniques and subsequently cloned into the plasmid C-YC426ADH^48^ using BamHI and HindIII or EcoRI and HindIII restriction sites, respectively. The plasmid C-YN425ADH was used as a template for the PCR amplification of the YFP(N) fragment. The PCR product was inserted into pGEM4 using KpnI and BamHI restriction sites. The DHFR gene in pYX142-DHFR, pYX142-cp(VDAC)-DHFR and pYX142-hp18(DHFR) was then replaced with the YFP(N) fragment by sub-cloning with NcoI and SalI restriction sites.

The plasmid pGEM4-hVDAC1 was used as a template for the PCR amplification of the full-length and the truncated hVDAC1 variants. The amplification products were inserted into pYX142 using BamHI and SalI restriction sites.

All DNA sequences required for the generation of constructs for the self-assembly-GFP assays were amplified from *Arabidopsis thaliana* Col0 or from *Pisum sativum* var. arvika cDNA, by standard PCR techniques. Subsequently the PCR products were cloned into the pAVA plasmid^49^ containing the fragments for saGFP11 (GFP_S11_) (N-terminally) or saGFP1-10 (GFP_S1–10_). Templates for the saGFP_S1–10_ and saGFP-_S11_ fragments were obtained from Dr G. S. Waldo (Los Alamos, NM, USA). Chimeric β-barrel proteins were generated using PCR and were cloned into the saGFP-_S11_ (N-terminally) pAVA vector using KpnI and SpeI as restriction sites. All plasmids used in this study are listed in Supplementary Table 2.

### Biochemical procedures

Mitochondria were isolated from yeast cells by differential centrifugation as described^50^. For the isolation of crude mitochondrial fractions, yeast cells were grown in selective media to logarithmic phase and harvested by centrifugation. The cells were resuspended in SEM buffer (250 mM sucrose, 10 mM MOPS, 1 mM EDTA, pH 7.4) supplemented with 2 mM Phenylmethylsulfonyl fluoride (PMSF), mixed with glass beads and lysed by three rounds of 30 s vortexing followed by 30 s on ice. The whole-cell lysate was separated from glass beads and cell debris by centrifugation (1,000*g*, 3 min, 2 °C). Crude mitochondria were isolated from the cell lysate by centrifugation (20,000*g*, 10 min, 2 °C). In addition, a sample from the whole-cell lysate was subjected to chloroform–methanol precipitation. The samples were then subjected to SDS–PAGE.

Radiolabelled proteins were synthesized in rabbit reticulocyte lysate in the presence of ^35^S-methionine (Perkin-Elmer) after *in vitro* transcription by SP6 polymerase from pGEM4 vectors (Promega). Radiolabelled precursor proteins were incubated at 25 °C with isolated yeast mitochondria in import buffer (250 mM sucrose, 0.25 mg ml^−1^ BSA, 80 mM KCl, 5 mM MgCl_2_, 10 mM MOPS-KOH, 2 mM NADH, 4 mM ATP, pH 7.2). Non-imported proteins were removed by treatment with PK (50 μg ml^−1^) for 30 min on ice. After inhibition of PK with 5 mM PMSF, the samples were boiled at 95 °C for few min before their analysis by SDS–PAGE.

### Immunoblotting

A list of primary antibodies used in this study is included in Supplementary Table 3. The secondary antibodies were Horseradish peroxidase-coupled goat-anti-rabbit or goat-anti-mouse (Bio-Rad, cat. # 1721019 or cat. # 1721011, respectively) that were diluted 1:10,000 or 1:2,000, respectively. Full uncropped versions of all immunoblot images in all figures are included in Supplementary Fig. 9.

### Synthesis and purification of peptides

The linear protected peptide was synthesized using standard Fmoc/tBu chemistry on a multiple peptide synthesizer Syro II (MultiSyntechTech) on an acid labile TCP resin (Intavis) as described^51^. To remove the peptide from the resin after completion of the synthesis, the dried resin was treated for 2 h at room temperature with a mixture of dichloromethane/1,1,1,3,3,3-hexafluoro-2-propanol (8/2 v/v). The mixture was filtered out and the solvent was concentrated with vacuum. The obtained oily product was precipitated from cold diethylether as a white solid. The linear protected peptide was treated with trifluoroacetic acid (TFA) and purified by RP-HPLC as described below. The Matrix-Assisted Laser Desorption Ionization-Time of Flight-Mass Spectrometry (MALDI-TOF-MS) showed the correct mass of [M+H]^+^=2,454.50.

A cyclic protected peptide was obtained as follows. Diisopropylethylamine (0.6 mmol) and 1-hydroxybenzotriazol (0.3 mmol) were added to a solution of linear protected peptide (0.1 mmol) in dry 40 ml dimethylformamide. The solution was dropped to a solution of 0.3 mmol *O*-benzotriazol-1-yl-N,N,N′N′-tetramethyluronium tetrafluoroborate in 80 ml dimethylformamide for 2 h, and the solution was stirred overnight. The solvent was removed under reduced pressure affording a light yellow oily residue. The crude cyclic product was then precipitated from ice-cold water and was dried under vacuum for 12 h.

Preparation of free cyclic peptide: The dried residue was treated with a mixture of TFA/thioanisole/water (95:5:5) for 3 h at room temperature and the final cyclic peptide was precipitated as an amorphous solid by the addition of diethylether. Crude peptides were purified further by preparative RP-HPLC on a Reprosil-Pur Basic C8, 5 μm column (250 × 10 mm) (Dr Maisch, Germany) using a linear gradient of 20–70% acetonitrile in water containing 0.05% TFA. Purification was verified by analytical RP-HPLC. The cyclization was confirmed by MALDI-TOF-MS by obtaining the correct mass [M+H]^+^=2,436.48.

### Modelling and prediction of secondary structures

The secondary structures of atVDAC1 and scPorin were assigned based on an alignment to the crystal structure of mouse VDAC1 (programme: Mafft v6.847b, options: local pair, JTT200 matrix, 2.65 gap open, 0.15 gap extension^52^). For psOEP24 the secondary structure elements were predicted using the consensus prediction of the GeneSilico metaserver^53^, the BOCTOPUS server^54^ and the PredTMbb server^55^. For each position a score describing the likelihood of this residue to be part of a β-strand was calculated as follows: the score for each position was increased by 1 if BOCTOPUS and PredTMbb predict the corresponding position as part of a β-strand and by 0.5 if the secondary structure prediction of GeneSilico metaserver predicts β-strand. The prediction of the GenSilico metaserver had a lower weight because this server predicts β-strands in general whereas the other two predict transmembrane β-strands. Furthermore the score was decreased by 0.5 if the position was assigned as disordered by the GeneSilico metaserver. A residue was assigned to be in a β-strand if its score was higher than 1.5. To avoid missing potential β-strands we additionally checked manually an alignment of OEP24 sequences from several organisms for regions of alternating hydrophobicity^56^,^57^.

### Protoplast transfection and fractionation

*A. thaliana* protoplasts were isolated and transfected as described^29^. Two samples of 2.5 million protoplasts each were transfected with 200 μg plasmid DNA of GFP_S11_-OEP24_1–12_-VDAC1_18–19_ and 150 μg plasmid DNA of YC3.60-TOM20. After 14 h expression under constant light, protoplasts were collected at 100*g* for 5 min and pooled in 5 ml extraction buffer (0.3 M sucrose, 50 mM HEPES-KOH, pH 7.6, 2 mM EDTA, 1% (w/v) polyvinylpyrrolidone (PVP) 40, 1% (w/v) fatty acid-free BSA, 330 mg l^−1^ ascorbate, supplemented immediately before use with 2 mM PMSF). For the total protein extract, 500 μl of protoplast suspension were pelleted at 25,000*g* for 5 min and resuspended in loading buffer (8 M urea, 0.2 M Tris–HCl pH 6.8, 10 mM EDTA pH 8.0, 5% (w/v) SDS, 0.03% bromphenol blue, 10 mM PMSF and 143 mM β-mercaptoethanol). The protoplasts were lysed using an Ultra-Turrax T25 homogenizer for three times of one second each at low intensity (idling speed=8,000 min^−1^). The lysate was then aliquoted in 1.5 ml Eppendorf tubes and the crude chloroplasts fraction was harvested at 1,500*g* for 5 min. The pellets were resuspended in a total volume of 2.5 ml washing buffer (0.3 M sucrose, 50 mM HEPES-KOH pH 7.6, 10 mM MgCl_2_, 10 mM KCl, 2 mM EDTA pH 8.0, 330 mg l^−1^ ascorbate, supplemented immediately before use with 1 mM PMSF). Chloroplasts were purified over a Percoll step gradient (3 ml 85% Percoll and 5.143 ml 45% Percoll both diluted in 0.33 M sorbitol and 50 mM HEPES-KOH, pH 7.6) and washed twice with washing buffer at 1,500*g* for 2 min. The final pellets were pooled in a total volume of 500 μl chloroplast-treatment buffer (0.33 M sorbitol, 50 mM Hepes KOH pH 7.6 and 5 mM MgCl_2_).

The supernatants of the crude chloroplasts fraction were centrifuged at 1,500*g* for 5 min. The resulting supernatants were centrifuged at 25,000*g* for 15 min to collect the mitochondrial fraction. The pellets were pooled in a total volume of 500 μl mitochondria-treatment buffer (0.3 m sucrose, 50 mM HEPES-KOH pH 7.6, 10 mM MgCl_2_ and 10 mM KCl). Chloroplast and mitochondrial fractions were portioned into five 100 μl aliquots and the organelles were pelleted again (chloroplasts: 1,500*g*, 5 min; mitochondria: 25,000*g*, 15 min). The purity of the organelles was probed by western blot analysis with an antibody against full-length GFP (Roche) to verify that YC3.60-Tom20 is solely found in the mitochondria enriched fraction. Enrichment of plastidic fraction is shown by DB71 staining of RUBISCO. A fraction of each organelle (100 μl) was subjected to protease treatment (chloroplasts: 120 μg ml^−1^ thermolysin; mitochondria: 5 μg ml^−1^ proteinase K^30^) in the respective treatment buffer. After protease treatments the organelles were pelleted again (chloroplasts: 1,500*g*, 5 min; mitochondria: 25,000*g*, 15 min). Additional two fractions of each organelle (100 μl each) were incubated on ice for 30 min with either 100 mM Na_2_CO_3_, pH 11.5 or with 1% Triton X-100. All samples including the untreated control samples were centrifuged at 100,000*g* for 30 min at 4 °C and proteins of the pellet were analyzed by SDS–PAGE followed by the western blot analysis with antibodies against GFP_S11_ (Peptide Speciality Laboratories, Antigen: RDHMVLHEYVNAAGIT-C) or full-length GFP to detect the fusion proteins.

### Fluorescence microscopy

For analyzing protoplasts by fluorescence microscopy GFP fluorescence, chlorophyll autofluorescence and the MitoTracker signal were monitored by confocal laser scanning microscopy using a TCS SP5 microscope (Leica) with an HCX PL APO Lambda Blue 63 × 1.4 oil objective. Fluorescence was excited and detected as follows: GFP 488/505–525 nm, chlorophyll fluorescence 514/650–750 nm and MitoTracker Orange CNTM Ros 554/576 nm. Images were processed by Leica LAS AF Lite Software.

### BIFC assay

Yeast cells co-expressing either Tom20-YFP(C) or Mcr1(R4E, R7E)-YFP(C) and YFP(N) fusion proteins were grown in selective liquid medium to logarithmic phase and were used for fluorescence microscopy with an Axioskop20 fluorescence microscope equipped with an Axiocam MRm camera. For quantitative analysis, crude mitochondria were isolated from the cells and were then resuspended in SEM buffer and analyzed for their YFP fluorescence using a Tecan infinite 200 microplate reader.

### Photo-crosslinking

*In organello* photo cross-linking was performed by mixing isolated mitochondria at a final concentration of 1 mg ml^−1^ with the Bpa-containing linear or cyclic peptide in import buffer without BSA. The mixture was incubated for 10 min on ice before ultravoilet-irradiation for 30 min at 4 °C. For irradiation, a Blak-Ray B-100 AP ultravoilet lamp at a distance of 10 cm from the samples was used. After the ultravoilet-illumination, the mitochondria were re-isolated by centrifugation (20,000*g*, 10 min, 2 °C) and subjected to SDS–PAGE.

### NMR analysis and structural assignment

Samples of the two peptides (1 mg peptide, ∼903 μM) were prepared in peptide buffer (10 mM 3-(N-morpholino)propanesulfonic acid, 80 mM KCl, 5 mM MgCl_2_, pH 7.2 in 11% (v/v) dimethylsulfoxid (DMSO)) and 10% (v/v) D_2_O was then added. The measurements were carried out on a Bruker Avance 800 MHz spectrometer equipped with a triple resonance cryogenic probe. For assignment of the HN and Hα resonances, ^1^H, ^1^H NOESY spectra with a WATERGATE water suppression scheme and a mixing time of 200 ms and two ^1^H, ^1^H TOCSY spectra with mixing times of 15 and 60 ms, respectively, were recorded at 5 °C. Spectra were processed and analyzed using Bruker TopSpin 2.1/3.2.

For studying the interaction of the β-hairpin peptide with dTom20 the cyclic peptide and uniformly [^15^N]-labelled rat dTom20 ([^15^N]-dTom20) were used. Preparation of [^15^N]-dTom20 was performed as reported previously^39^ with minor modifications. The gene for the cytosolic receptor domain of dTom20 from *Rattus norvegicus* (residues 51–145) lacking the stop codon was cloned into pET-22b (Merck Millipore) for expression of the fusion protein with a hexa-histidine tag at the C terminus. The *E. coli* strain BL21(DE3) transformed with this plasmid was cultured in M9 media (1 g l^−1^ of [^15^N]-enriched NH_4_Cl, 18 g l^−1^ of Na_2_HPO_4_ × 12H_2_O, 3 g l^−1^ of KH_2_PO_4_, 2 g l^−1^ D-glucose, 1 mM MgSO_4_ × 7H_2_O, 0.1 mM CaCl_2_, 10 mg l^−1^ thiamin) containing 50 μg ml^−1^ ampicillin at 37 °C until OD_600_ reached 0.5. Protein expression was induced by 0.5 mM isopropyl β-D-1-thiogalactopyranoside for 16 h at 16 °C. Then cells were collected by centrifugation and re-suspended in 20 mM Tris–HCl, pH 7.4, containing 300 mM NaCl followed by cell disruption by sonication. The cell lysate was subjected to centrifugation to remove cell debris and unbroken cells and the supernatant was loaded onto a Ni-NTA column (QIAGEN) for affinity purification. Eluted proteins were further purified by gel-filtration chromatography on a HiLoad 26/600 Superdex 200 pg column (GE Healthcare). Fractions containing [^15^N]-dTom20 were pooled and dialyzed against 20 mM KPi, pH 6.4, containing 50 mM KCl and stored at 4 °C until use. NMR spectra were recorded on a Bruker AVANCE900 spectrometer equipped with a TCI cryogenic probe. For NMR titration experiments, aliquots of 10 mM β-hairpin peptide in DMSO were added to 210 μM [^15^N]-dTom20 in 20 mM KPi, pH 6.4, 50 mM KCl, 5% (v/v) DMSO, D_2_O/H_2_O (5/95). To subtract the solvent effects of DMSO from chemical shift perturbation by the β-hairpin peptide, an NMR spectrum of [^15^N]-dTom20 with 9% DMSO, the final DMSO concentration in the titration experiments, was also recorded with the assumption that the solvent effect is linear to the added solvent volume. The chemical shift changes of each backbone amide of dTom20 were calculated according to the equation [(Δ*δ*(^1^H_p_−^1^H_D_)^2^+Δ*δ*((^15^N_p_−^15^N_D_)/5)^2^]^1/2^, where Δ*δ*(^1^H_p_−^1^H_D_) and Δ*δ*(^15^N_p_−^15^N_D_) are the net chemical shift differences for the peptide after subtraction of the effect of DMSO.

### Data availability

The authors declare that all data supporting the findings of this study are available within the article and its Supplementary Information files or are available from the corresponding author upon request.

## Additional information

**How to cite this article:** Jores, T. *et al.* Characterization of the targeting signal in mitochondrial β-barrel proteins. *Nat. Commun.* 7:12036 doi: 10.1038/ncomms12036 (2016).

## Supplementary Material

Supplementary Information Supplementary Figures 1 - 9, Supplementary Tables 1 - 3 and Supplementary References
