# Supplementary material for: Characterization of the targeting signal in mitochondrial β-barrel proteins
Source: Nat Commun. 2016 Jun 27;7:12036. doi: 10.1038/ncomms12036 (PMC4931251; doi:10.1038/ncomms12036)
Supplement: Supplementary Information — Supplementary Figures 1 - 9, Supplementary Tables 1 - 3 and Supplementary References [file ncomms12036-s1.pdf]

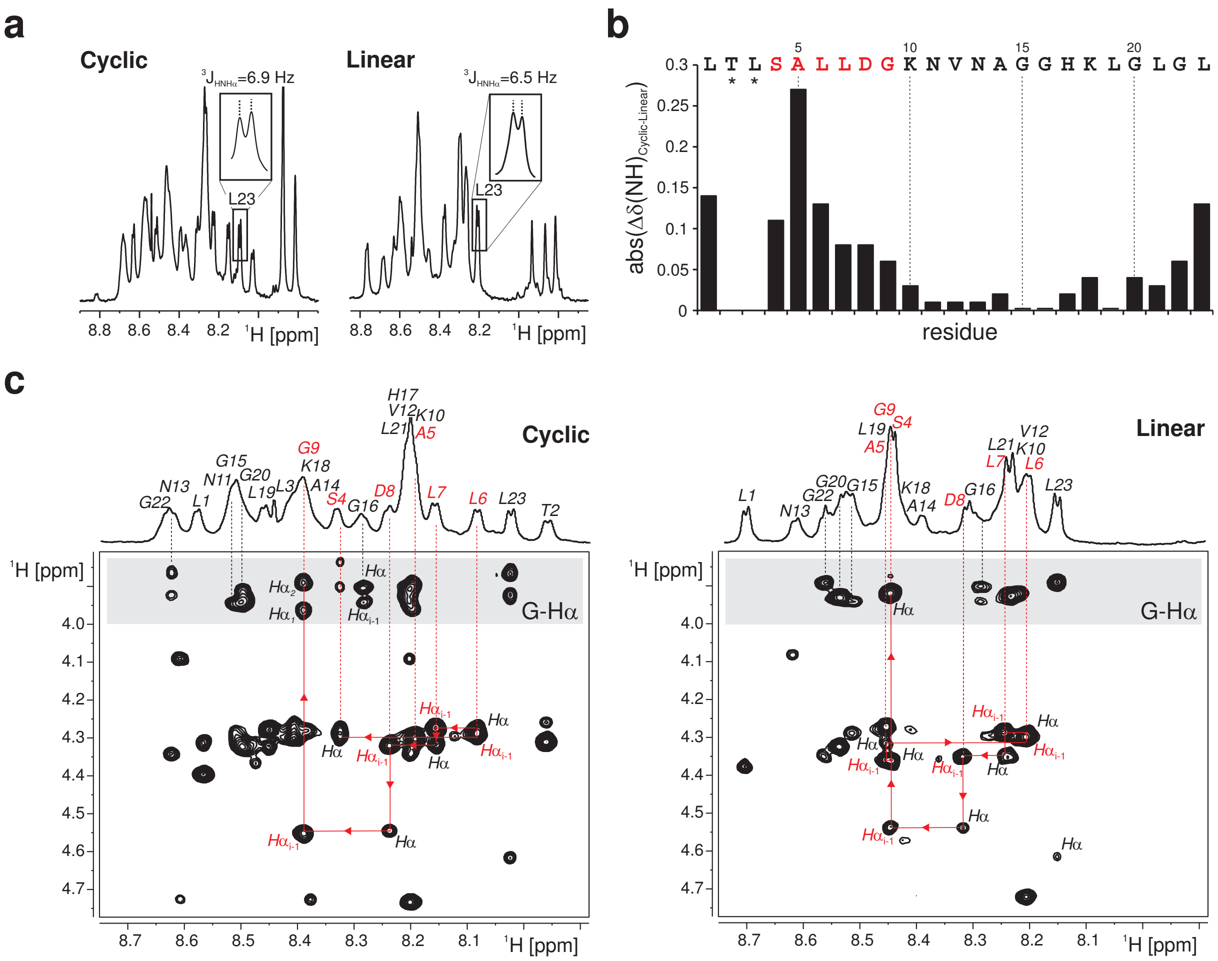

Supplementary Figure 1 | Structural analysis by NMR of the two peptides used for import competition. (**a**) Amide region of 1D ^{1}H spectra of the cyclic (left) and the linear (right) peptide at 10 °C. The inset shows the amide resonance of L23. The ^{3}J(HN,Hα) couplings splitting these resonances are indicated. (**b**) Amide proton chemical shift differences between the cyclic and the linear peptide at 5 °C. The sequence stretch, for which the sequential assignment is shown in (c), is highlighted in red. Residues, for which the difference could not be determined due to a lack in NH assignment, are labeled with an asterisk. (**c**) HN-Hα region of 2D ^{1}H,^{1}H NOESY spectra of the cyclic (left) and the linear (right) peptides at 5 °C. The assigned 1D ^{1}H spectrum of the amide region is shown on top. Intraresidual (HN-Hα$_i$, labeled in black) and sequential (HN-Hα$_{i-1}$, labeled in red) connections are indicated for the sequence stretch S4 to G9. The spectral region typical of glycine HN-Hα cross peaks residues is labeled in grey.

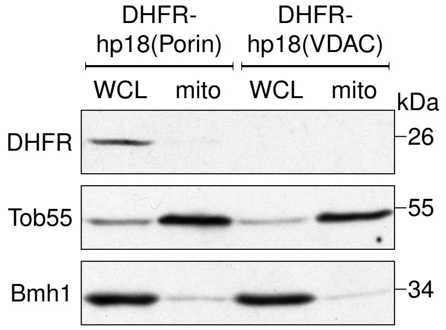

Supplementary Figure 2 | Fusion proteins with a β-hairpin fused C-terminally to DHFR are not targeted to mitochondria. Crude mitochondria were isolated from yeast cells expressing DHFR alone or the indicated fusion proteins. Samples from the whole cell lysate (WCL) and the crude mitochondria (mito) were analyzed by SDS-PAGE and immunodecoration with antibodies against the indicated proteins. Tob55, mitochondrial β-barrel protein; Bmh1, cytosolic protein.

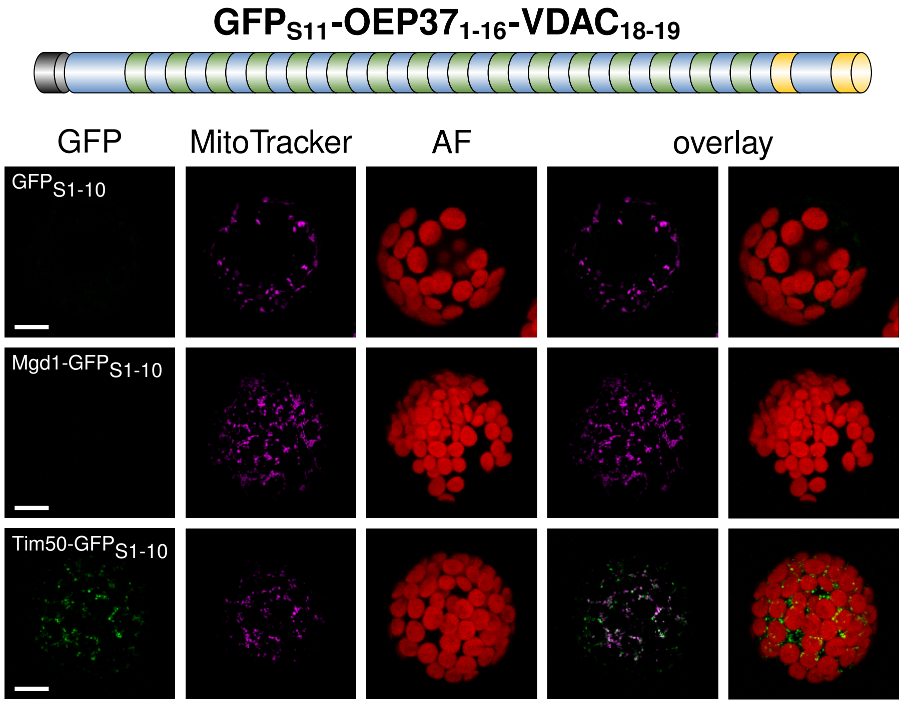

Supplementary Figure 3 | The last β-hairpin of atVDAC1 directs psOEP37 to mitochondria. GFP_{S11}-$OEP37_{1\text{-}16}$-$VDAC_{18\text{-}19}$ and the indicated reporter constructs were co-transformed into *A. thaliana* protoplasts. Signals and schemes are as in Fig. 3a. Scale bar: 10 µm.

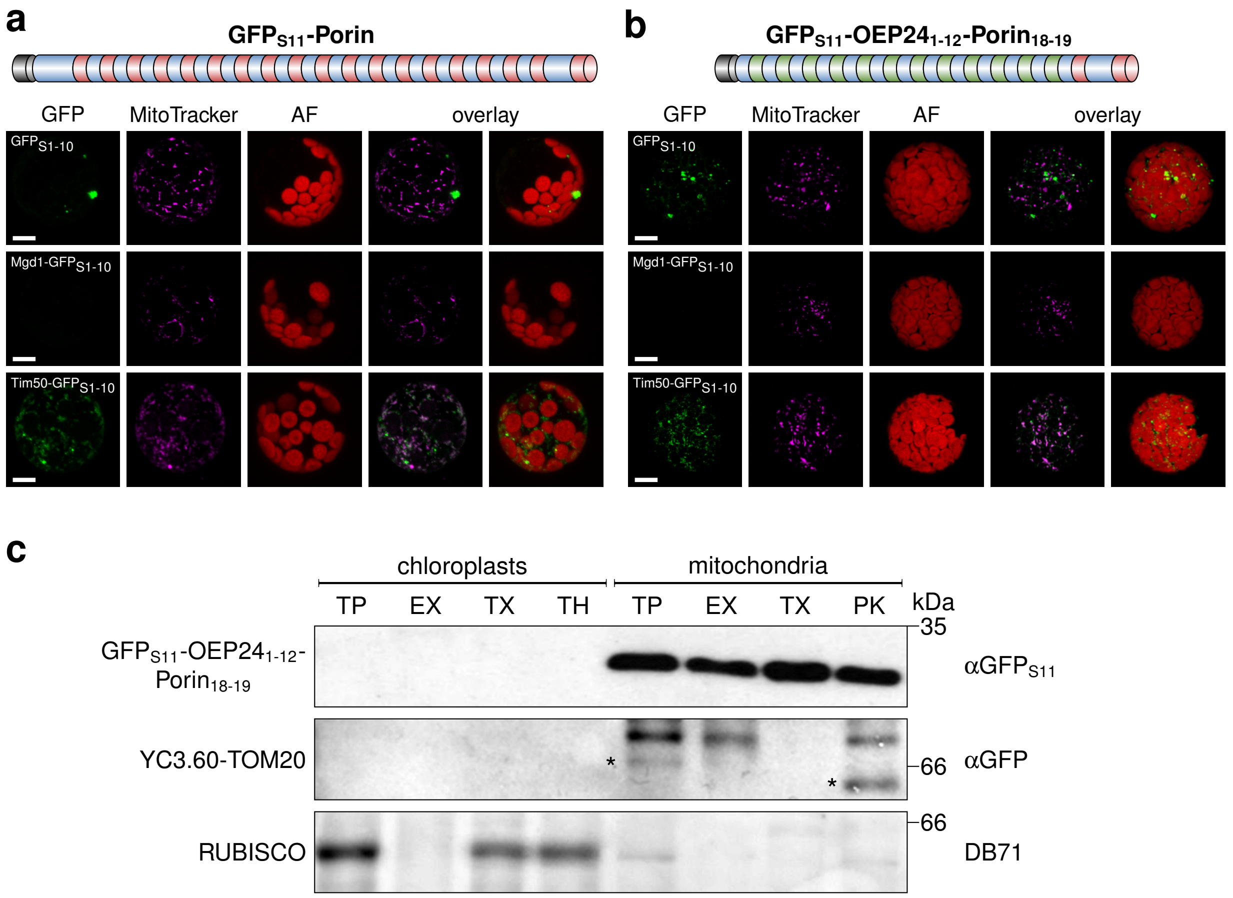

Supplementary Figure 4 | The last β-hairpin of scPorin targets psOEP24 to mitochondria. (**a** and **b**) GFP_{S11}-Porin (**a**) or GFP_{S11}-$OEP24_{1\text{-}12}Porin_{18\text{-}19}$ (**b**) and the indicated reporter constructs were co-transformed into *A. thaliana* protoplasts. Images were taken as described in the legend to Fig. 3a. Schemes of scPorin and the construct generated are shown. Red/green sections indicate transmembrane β-sheets of scPorin and psOEP24, respectively. The black section represents the GFP_{S11}-tag. Scale bar: 10 µm. (**c**) Organelles were carbonate extracted (EX), solubilized by addition of Triton X-100 (TX), or treated with either thermolysin (TH) or proteinase K (PK). Further treatment and analysis were as described in the legend to Figure 3b. Bands resulting from cross-reactivity of the GFP antibody are marked with an asterisk.

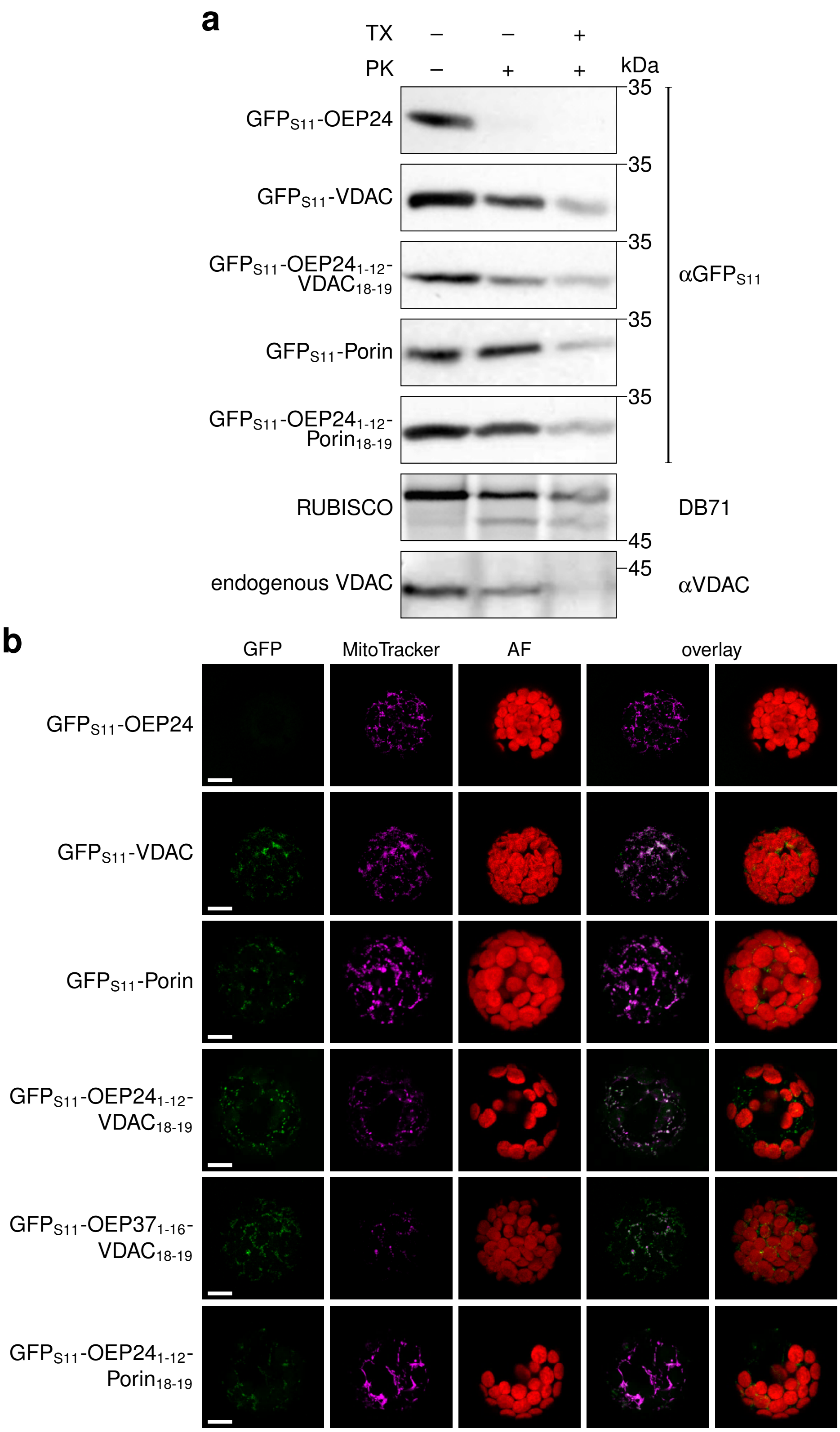

Supplementary Figure 5 | The last β-hairpin of atVDAC1 or scPorin targets chloroplast β-barrel proteins to the mitochondrial intermembrane space. (**a**) Plasmids coding for the indicated GFP_{S11}-tagged proteins were transformed individually into *A. thaliana* protoplasts. Protoplasts were lysed and incubated with proteinase K (PK, 5 µg/mL final concentration) and Triton X-100 (TX, 1 % final) where indicated. After incubation, the cell lysate was subjected to SDS-PAGE followed by Western blotting with antibodies against GFP_{S11}. DB71 staining was used to visualize RUBISCO and immunodecoration with antibodies against the endogenous mitochondrial protein atVDAC1 was used to confirm the experimental procedure. (**b**) The indicated proteins and the reporter construct Tim21(N)-$GFP_{S1\text{-}10}$ were co-transformed into *A. thaliana* protoplasts. Images were taken as described in the legend to Fig. 3a. Scale bar: 10 µm.

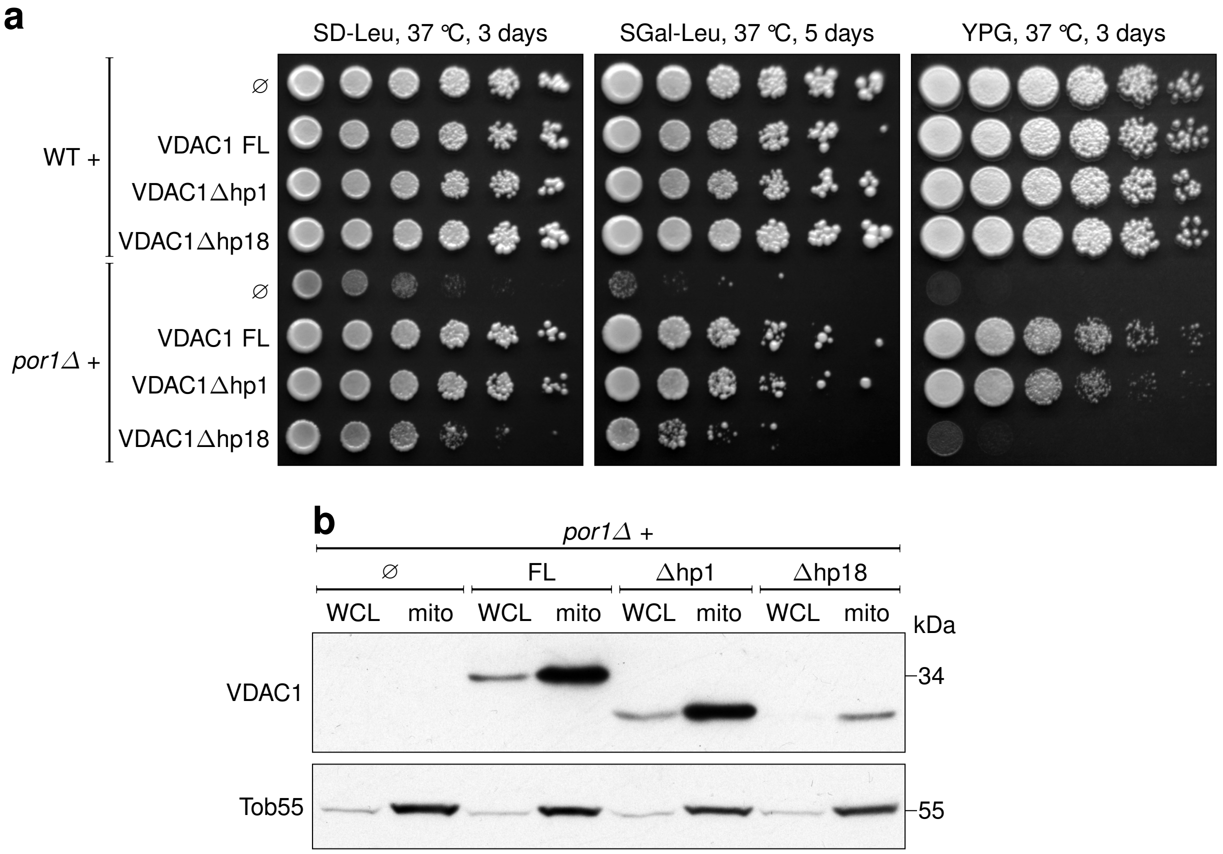

Supplementary Figure 6 | The last β-hairpin of atVDAC1 is important for mitochondrial targeting. (**a**) WT and *por1∆* cells were transformed with an empty plasmid (∅) or with a plasmid encoding either full-length VDAC1 (VDAC1 FL) or a truncated VDAC1 variant where the first (VDAC1∆hp1) or last (VDAC1∆hp18) β-hairpin was deleted . The growth of the cells at elevated temperature (37 °C) was analyzed by drop-dilution assay on the indicated media. (**b**) Crude mitochondria from the *por1∆* cells described in (a) were isolated. The whole cell lysate (WCL) and the mitochondrial fraction (mito) were subjected to SDS-PAGE and immunoblotting with antibodies against VDAC1. Tob55 was used as a loading control.

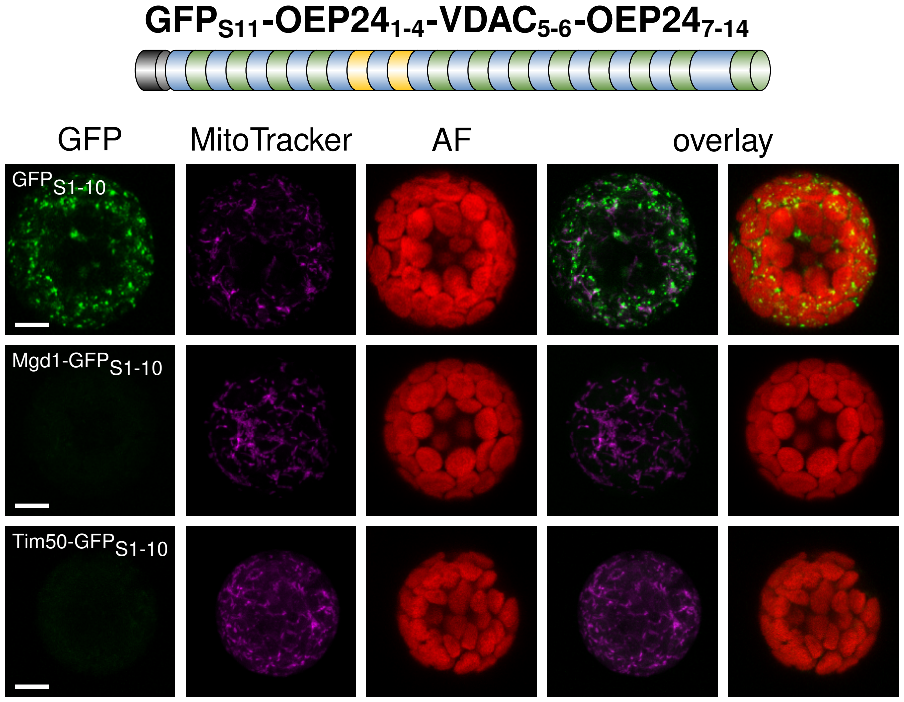

Supplementary Figure 7 | The fifth and sixth β-strands of atVDAC1 do not direct psOEP24 to mitochondria. The indicated fusion protein and reporter constructs were co-transformed into *A. thaliana* protoplasts. Signals and schemes are as in Fig. 3a. Scale bar: 10 µm.

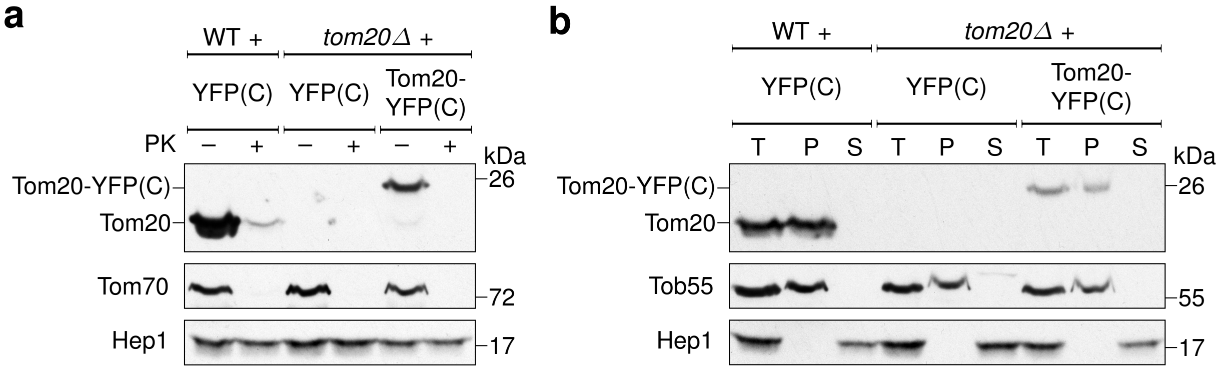

Supplementary Figure 8 | Tom20-YFP(C) is imported into the outer membrane of mitochondria in a native-like conformation. (**a**) Isolated mitochondria from either WT or *tom20Δ* strains expressing the C-terminal part of YFP (YFP(C)) or Tom20 fused N-terminally to YFP(C) were treated with 50 µg/mL proteinase K (PK) or left untreated. The samples were analyzed by SDS-PAGE and immunodecoration. Tom70, outer membrane protein exposed to the cytosol; Hep1, matrix protein. (**b**) Carbonate extraction of mitochondria isolated from the strains described in (a) was performed to separate membrane-embedded proteins in the pellet fraction (P) from soluble protein in the supernatant (S). T, total; Tob55, membrane-embedded β-barrel protein; Hep1, soluble matrix protein.

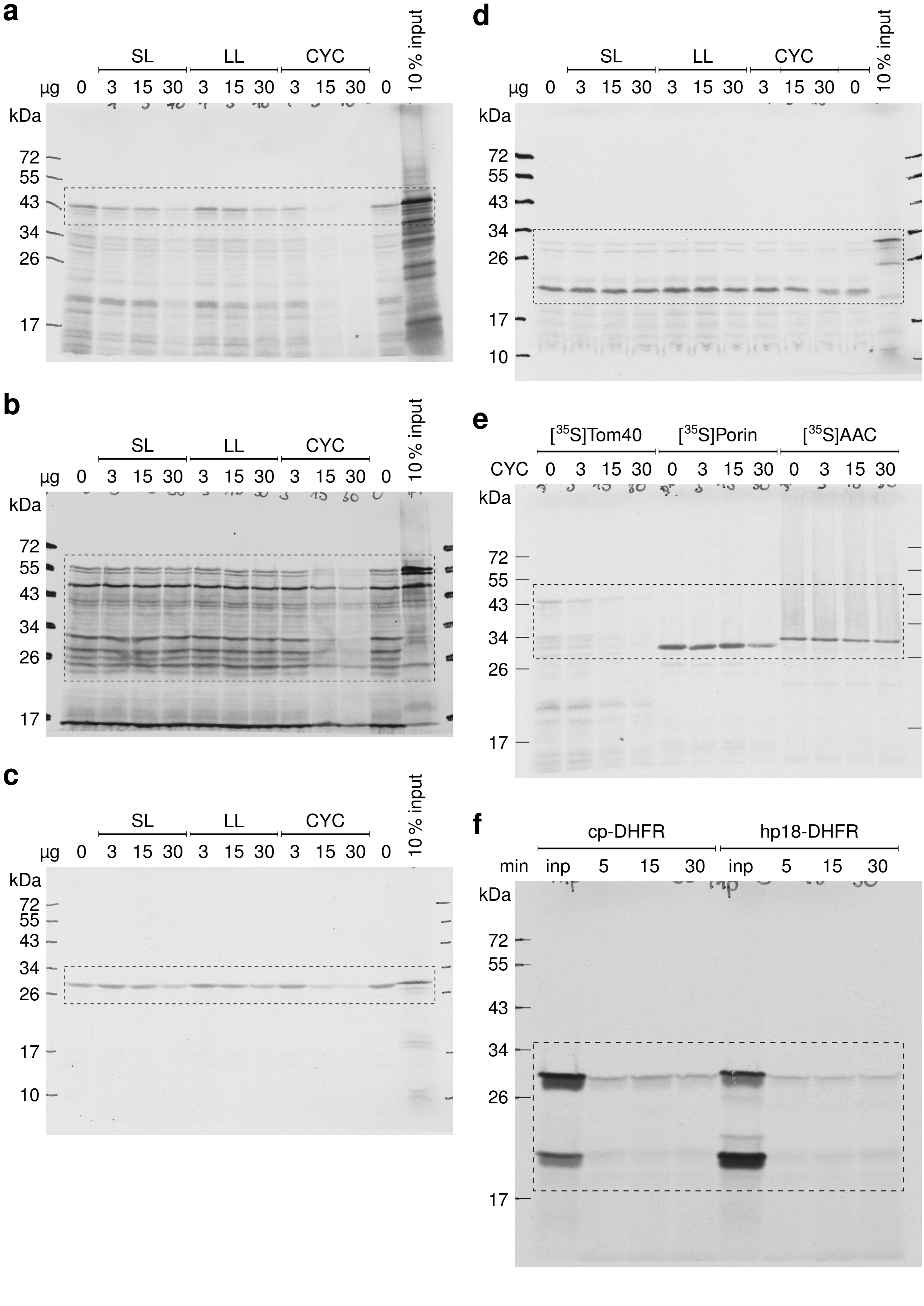
a
SL
LL
CYC
μg 0 3 15 30 3 15 30 3 15 30 0
10 % input
kDa
72
55
43
34
26
17
b
SL
LL
CYC
μg 0 3 15 30 3 15 30 3 15 30 0
10 % input
kDa
72
55
43
34
26
17
c
SL
LL
CYC
μg 0 3 15 30 3 15 30 3 15 30 0
10 % input
kDa
72
55
43
34
26
17
10
d
SL
LL
CYC
μg 0 3 15 30 3 15 30 3 15 30 0
10 % input
kDa
72
55
43
34
26
17
10
e
[35S]Tom40
[35S]Porin
[35S]AAC
CYC 0 3 15 30 0 3 15 30 0 3 15 30
kDa
72
55
43
34
26
17
f
cp-DHFR
hp18-DHFR
min inp 5 15 30 inp 5 15 30
kDa
72
55
43
34
26
17

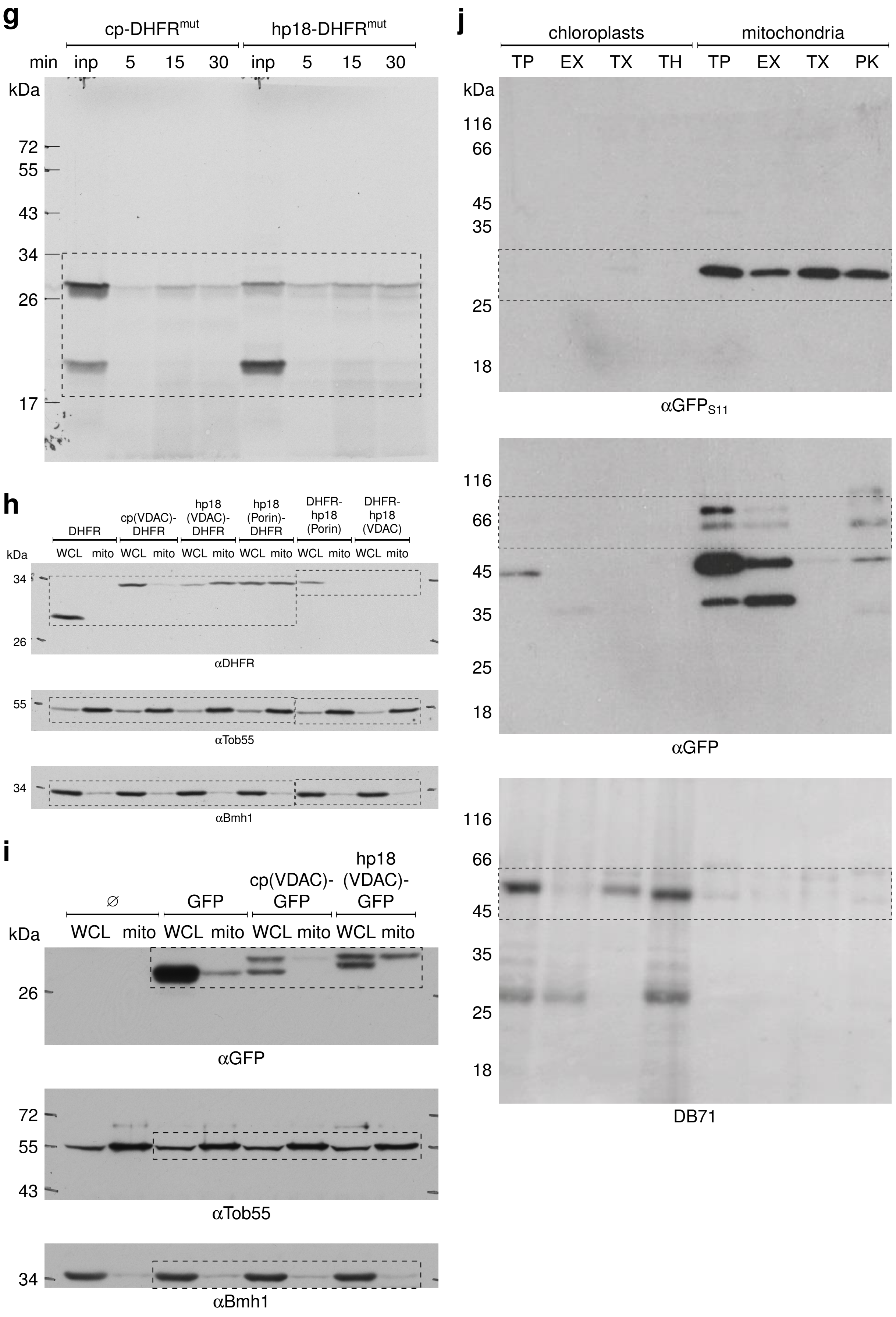
g
cp-DHFRmut
hp18-DHFRmut
min inp 5 15 30 inp 5 15 30
kDa
72
55
43
34
26
17
h
DHFR
cp(VDAC)-DHFR
hp18 (VDAC)-DHFR
hp18 (Porin)-DHFR
DHFR-hp18 (Porin)
DHFR-hp18 (VDAC)
WCL mito WCL mito WCL mito WCL mito WCL mito WCL mito
kDa
34
26
αDHFR
55
αTob55
34
αBmh1
i
∅
GFP
cp(VDAC)-GFP
hp18 (VDAC)-GFP
WCL mito WCL mito WCL mito WCL mito
kDa
26
αGFP
72
55
43
αTob55
34
αBmh1
j
chloroplasts
mitochondria
TP EX TX TH TP EX TX PK
kDa
116
66
45
35
25
18
αGFPS11
116
66
45
35
25
18
αGFP
116
66
45
35
25
18
DB71

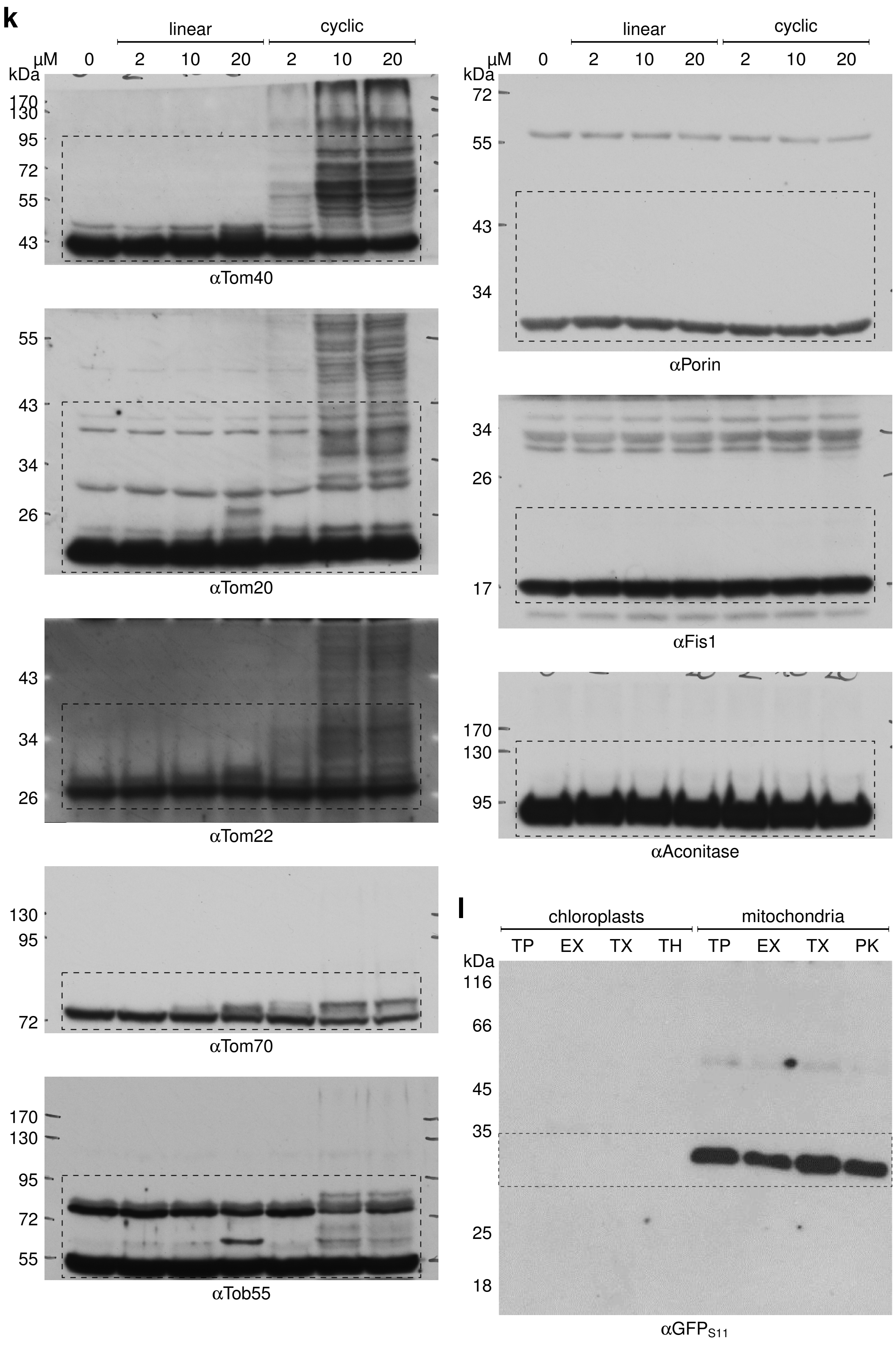
k
linear
cyclic
µM
0
2
10
20
2
10
20
kDa
170
130
95
72
55
43
αTom40
55
43
34
26
αTom20
43
34
26
αTom22
130
95
72
αTom70
170
130
95
72
55
αTob55
72
55
43
34
αPorin
34
26
17
αFis1
170
130
95
αAconitase
l
chloroplasts
mitochondria
TP
EX
TX
TH
TP
EX
TX
PK
kDa
116
66
45
35
25
18
αGFP$_{S11}$

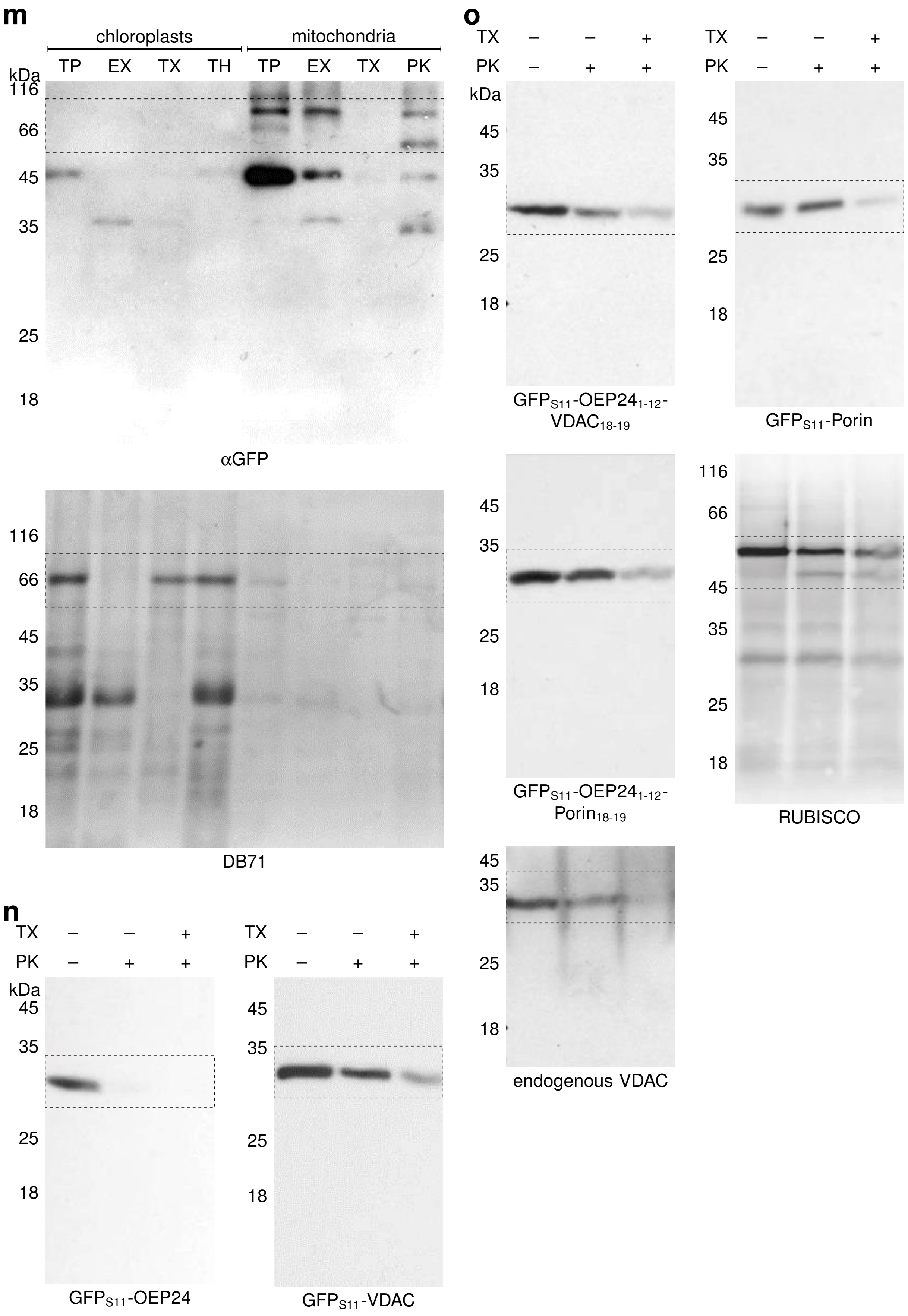
m
chloroplasts
mitochondria
TP EX TX TH TP EX TX PK
kDa
116
66
45
35
25
18
αGFP
116
66
45
35
25
18
DB71
n
TX – – +
PK – + +
kDa
45
35
25
18
GFP$_{S11}$-OEP24
TX – – +
PK – + +
45
35
25
18
GFP$_{S11}$-VDAC
o
TX – – +
PK – + +
kDa
45
35
25
18
GFP$_{S11}$-OEP24$_{1-12}$-VDAC$_{18-19}$
TX – – +
PK – + +
45
35
25
18
GFP$_{S11}$-Porin
45
35
25
18
GFP$_{S11}$-OEP24$_{1-12}$-Porin$_{18-19}$
116
66
45
35
25
18
RUBISCO
45
35
25
18
endogenous VDAC

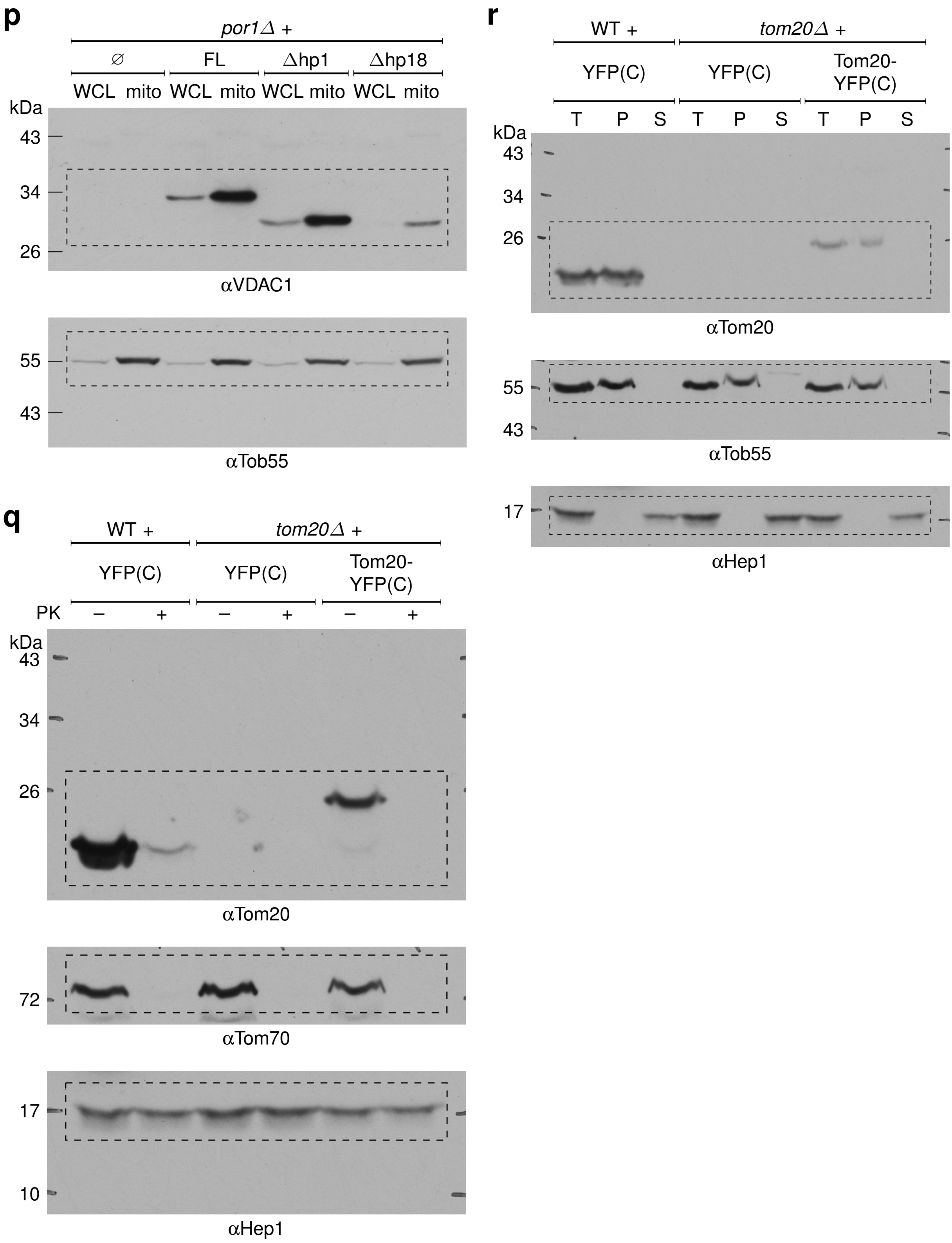

Supplementary Figure 9 | Full immunoblots and autoradiography films. (**a**-**r**) Original images of the blots shown in Figs. 1b (**a**), 1c (**b**), 1d (**c**), 1e (**d**), 1f (**e**), 2a (**f** and **g**), 2c (**h**), 2f (**i**), 3e (**j**), 7 (**k**) and in Supplementary Figs. 2 (**h**), 4c (**l** and **m**), 5a (**n** and **o**), 6b (**p**) 8a (**q**), and 8b (**r**). Cropped areas are marked by dashed lines.

Supplementary Table 1 | Primers used in this study.

Construct	Primer	Sequence (5' → 3')
GFP_{S11}-VDAC	atVDAC-1_FW	AATGGTACCATGGTGAAAGGTCCCGGTCTCTACACC
	atVDAC-1_RV	AATACTAGTAGGCTTGAGTGCGAGAGCCAATCC
GFP_{S11}-$OEP24_{1\text{-}10}$-$VDAC_{16\text{-}19}$	OEP+4VD_FW	CTACCTATGATGTTGCTCCCTTGACCTCTGTGAAGGC
	OEP+4VD_RV	CACAGAGGTCAAGGGAGCAACATCATAGGTAGGTTC
GFP_{S11}-$OEP24_{1\text{-}12}$-$VDAC_{18\text{-}19}$	OEP+2VD FW	GACAAGGAATTCCAAACCCAAGTCATTCTTCACAATC
	OEP+2VD_RV	GAAGAATGACTTGGGTTTGGAATTCCTTGTCCACTC
GFP_{S11}-$OEP24_{1\text{-}13}$-$VDAC_{**19}$	O-L1VD_RV	GTACACTAGTAGGCTTGAGTGCGAGAGCCAATCCAACTTTAGCACTCTTGTCAATTGACTTTGTGTTAACGGACGCCACAAC
GFP_{S11}-$OEP24_{1\text{-}13}$-$VDAC_{19}$	O-1VD_RV	CTAGTAGGCTTGAGTGCGAGAGCCAATCCAACTTTTTTGGGAATTTTC
GFP_{S11}-$OEP24_{1\text{-}4}$-$VDAC_{5\text{-}6}$-$OEP24_{7\text{-}14}$	O(1-4)-V(5-6)-O(7-14)_FW	TCTACTTTTCTGATCACCGCTACCGTTGATGAGGCTGCACCCGGACTGAGGTCAATCTTCAGCTTCAAGGTTCCTGAGAAACCGTTGAATTTGGC
	O(1-4)-V(5-6)-O(7-14)_RV	AGGAACCTTGAAGCTGAAGATTGACCTCAGTCCGGGTGCAGCCTCATCAACGGTAGCGGTGATCAGAAAAGTAGAGGGTTTCTCTACGGCGAGGACTAGGCC
GFP_{S11}-Porin	scPOR1Acc_FW	AATGGTACCATGTCTCCTCCAGTTTACAGCG
	scPOR1Bcu_RV	AATACTAGTAGCGTCGAAGGACAAAGACCAACC
GFP_{S11}-$OEP24_{1\text{-}12}$-$Porin_{18\text{-}19}$	O_2Por1_FW	CCTGGCGTCACTCTGGGTGTCGGTTCCTCTTTCGATGCTTTGAAGTTGTCTGAACCTGTTCACAAGCTAGGTTGGTCTTTGTCCTTCGACGCTACTAGTTAATCTAGAGTCCGCAAAAAT
	O_2Por1_RV	TAACTAGTAGCGTCGAAGGACAAAGACCAACCTAGCTTGTGAACAGGTTCAGACAACTTCAAAGCATCGAAAGAGGAACCGACACCCAGAGTGACGCCAGGTTGTTTGGAATTCCTTG
GFP_{S11}-$VDAC_{1\text{-}17}$-$OEP24_{13\text{-}14}$	VDAC+2O_FW	CAACACGAGTGGAAACAAACTGGTTGCTTCAAGGTTG
	VDAC+2O_RV	GAAGCAACCAGTTTGTTTCCACTCGTGTTGAATGAG
GFP_{S11}-$VDAC_{1\text{-}17}$-$OEP24_{13}VDAC1_{19}$	AK3-INT_FW	TCATTCTTCACAATCTCTGGAGAAGTCGACACAAAGTCAAAAATTCCCAAACTCAGTGTTGAGTCC
	AK3-INT_RV	GTCGACTTCTCCAGAGATTGTGAAGAATGACTTGGGTTTGGAATTCCTTGTCCACTCCAATCC
GFP_{S11}-$OEP37_{1\text{-}16}$-$VDAC_{18\text{-}19}$	OEP37_2VDAC1_FW	GGAGATGAAGGTGGGAAAGCATGGAAACCCAAGTCATTCTTCACAATCTCTGG
	OEP37_2VDAC1_RV	GATTGTGAAGAATGACTTGGGTTTCCATGCTTTCCCACCTTCATCTCCAACCCAAAGG
Tim21(N)-$GFP_{S1\text{-}10}$	Tim21-L1_KpnI_FW	AATGGTACCATGGTGAAACCATCGGATTTCAAAGC

Construct	Primer	Sequence (5' → 3')
	Tim21-L1_ TPTMD_SpeI_ RV	ATTACTAGTTGTAAACCTTGAGCGGAGGTATAATCC
DHFR	TJ009	CCCGGTACCATGGTTCGACCATTGAACTGC
	TJ011	CCCGGATCCTTAGTCTTTCTTCTCGTAGAC
DHFR^{mut}	TJ081	CCCGGTACCATGGTTCGACCATTGAAC
	TJ011	CCCGGATCCTTAGTCTTTCTTCTCGTAGAC
cp(VDAC)	TJ022	CCCGAATTCATGGCTGTGCCACCCACG
	TJ023	CCCGGTACCACCAGCACCAGCACCCAAATCAAGCTTTATTAAGCCAAATC
hp18(VDAC)	TJ014	CCCGAATTCATGAAGCCAGGTATTAAACTGAC
	TJ015	CCCGGTACCACCAGCACCAGCACCTGCTTGAAATTCCAGTCCTAGACC
hp18(Porin)	TJ012	CCCGAATTCATGAAGCAATTGTTAAGACCTGG
	TJ013	CCCGGTACCACCAGCACCAGCACCAGCGTCGAAGGACAAAGACC
hp2(Porin)	TJ060	CCCGAATTCATGAATGGCATTAAGTTCTCATTGAAG
	TJ061	CCCGGTACCACCAGCACCAGCACCGGTTTGCTTGTCATTCAACTTTG
hp3(Porin)	TJ037	CCCGAATTCATGAAAGACGGTCCACTGTC
	TJ038	CCCGGTACCACCAGCACCAGCACCGTTTGTGTTAGACCAGCC
hp17(VDAC)	TJ058	CCCGAATTCATGAACTCCAGCCTGATAGGTTTAG
	TJ059	CCCGGTACCACCAGCACCAGCACCGTTCTTGCCATCCAGAAGAG
hp3(VDAC)	TJ039	CCCGAATTCATGGAGACCACCAAAGTGAC
	TJ040	CCCGGTACCACCAGCACCAGCACCATTGTCGGTATTCCATTTCTC
GFP	TJ032	CCCGGTACCATGAGTAAGGGTGAAGAACTTTTCAC
	TJ034	CCCGGATCCTTATTTGTATAGTTCATCCATGCC
VDAC1 FL	TJ092	CCCGGATCCAATGGCTGTGCCACCCACG
	TJ093	CCCGTCGACTTATGCTTGAAATTCCAGTCCTAGAC
VDAC1Δhp1	TJ107	GGCTATGGATTTGGCACTGAGACCACCAAAGTGACG
	TJ108	CTTTGGTGGTCTCAGTGCCAAATCCATAGCCCTTGG
VDAC1Δhp18	TJ092	CCCGGATCCAATGGCTGTGCCACCCACG
	TJ094	CCCGTCGACTTATAGAGTCTGAGTGTATCCTAAACC
hp18(F281A)-DHFR	TJ014	CCCGAATTCATGAAGCCAGGTATTAAACTGAC
	TJ066	CCCGGTACCACCAGCACCAGCACCTGCTTGTGCTTCCAGTCCTAGACC
hp18(F281Q)-DHFR	TJ014	CCCGAATTCATGAAGCCAGGTATTAAACTGAC

Construct	Primer	Sequence (5' $\rightarrow$ 3')
	TJ046	CCCGGTACCACCAGCACCAGCACCTGCTTGTTGTTC CAGTCCTAGACC
hp18(L259Q, F281Q)-DHFR	TJ077	GTATTAAACTGACACAATCAGCTCTTCTG
	TJ078	CAGAAGAGCTGATTGTGTCAGTTTAATAC
hp18(L259Q, L277Q, F281Q)-DHFR	TJ079	ACAAGCTTGGTCAAGGACTGGAACAA
	TJ080	TTGTTCCAGTCCTTGACCAAGCTTGT
hp17(Q249L)-DHFR	TJ095	GTTTAGGATACACTTTGACTCTAAAGCC
	TJ096	GGCTTTAGAGTCAAAGTGTATCCTAAAC
hp17(Y247F, Q249L)-DHFR	TJ097	GATAGGTTTAGGATTCACTTTGACTCTAAAGCC
	TJ098	GGCTTTAGAGTCAAAGTGAATCCTAAACCTATC
Tom20-YFP(C)	TJ052	CCCGGATCCATGTCCCAGTCGAACCCTATC
	TJ053	CCCAAGCTTGTCATCGATATCGTTAGCTTCAG
Mcr1(R4E, R7E)-YFP(C)	TJ067	CCCGAATTCATGTTTTCCGAATTATCCGAATCTCACT CAAAAGC
	TJ068	CCCAAGCTTAAATTTGAAAACTTGGTCCTTGGAG
YFP(N)	TJ054	CCCGGTACCATGGTGAGCAAGGGCGAG
	TJ055	CCCGGATCCTCACTCGATGTTGTGGCG

Supplementary Table 2 | Plasmids used in this study.

Construct	Accession Number(s)	Reference
GFP$_{S11}$-OEP24	CAA04468	[1]
GFP$_{S11}$-VDAC1	AT3G01280	current study
GFP$_{S1\text{-}10}$		[2]
Mgd1-GFP$_{S1\text{-}10}$	AT4G31780	[2]
Tim50-GFP$_{S1\text{-}10}$	AT1G55900	[3]
Tim21(N)-GFP$_{S1\text{-}10}$	AT2G40800	current study
YC3.60-Tom20	AT3G27070	[1]
GFP$_{S11}$-OEP24$_{1\text{-}10}$-VDAC1$_{16\text{-}19}$	CAA04468 / AT3G01280	current study
GFP$_{S11}$-OEP24$_{1\text{-}12}$-VDAC1$_{18\text{-}19}$	CAA04468 / AT3G01280	current study
GFP$_{S11}$-OEP24$_{1\text{-}13}$-VDAC1$_{**19}$	CAA04468 / AT3G01280	current study
GFP$_{S11}$-OEP24$_{1\text{-}13}$-VDAC1$_{19}$	CAA04468 / AT3G01280	current study
GFP$_{S11}$-OEP24$_{1\text{-}4}$-VDAC1$_{5\text{-}6}$-OEP24$_{7\text{-}14}$	CAA04468 / AT3G01280	current study
GFP$_{S11}$-Porin	EDN62755	current study
GFP$_{S11}$-OEP24$_{1\text{-}12}$-Porin$_{18\text{-}19}$	CAA04468 / EDN62755	current study
GFP$_{S11}$-VDAC1$_{1\text{-}17}$-OEP24$_{13\text{-}14}$	CAA04468 / AT3G01280	current study
GFP$_{S11}$-VDAC1$_{1\text{-}17}$-OEP24$_{13}$-VDAC1$_{19}$	CAA04468 / AT3G01280	current study
GFP$_{S11}$-OEP37$_{1\text{-}16}$-VDAC1$_{18\text{-}19}$	CAB50915 / AT3G01280	current study
pGEM4-hVDAC1	P21796	[4]
pGEM4-Tom40	P23644	[5]
pGEM4-Tob55	P53969	[5]
pGEM4-Porin	EDN62755	[6]
pGEM4-pSu9-DHFR	P00842 / P00375	[7]
pGEM4-pSu9-DHFR^{mut}	P00842	[8]
pGEM4-cp(VDAC)-DHFR^{mut}	P21796	current study
pGEM4-hp18(VDAC)-DHFR^{mut}	P21796	current study
pGEM4-AAC	P04710	[9]
pYX142-DHFR	P00375	current study
pYX142-cp(VDAC)-DHFR	P21796 / P00375	current study
pYX142-hp18(VDAC)-DHFR	P21796 / P00375	current study
pYX142-hp18(F281A)-DHFR	P21796 / P00375	current study
pYX142-hp18(F281Q)-DHFR	P21796 / P00375	current study
pYX142-hp18(L259Q, F281Q)-DHFR	P21796 / P00375	current study

Construct	Accession Number(s)	Reference
pYX142-hp18(L259Q, L277Q, F281Q)-DHFR	P21796 / P00375	current study
pYX142-hp18(Porin)-DHFR	EDN62755 / P00375	current study
pYX142-hp2(Porin)-DHFR	EDN62755 / P00375	current study
pYX142-hp3(Porin)-DHFR	EDN62755 / P00375	current study
pYX142-hp17(VDAC)-DHFR	P21796 / P00375	current study
pYX142-hp17(Q249L)-DHFR	P21796 / P00375	current study
pYX142-hp17(Y247F, Q249L)-DHFR	P21796 / P00375	current study
pYX142-hp3(VDAC)-DHFR	P21796 / P00375	current study
pYX142-mtGFP		[10]
pYX142-eGFP		current study
pYX142-cp(VDAC)-eGFP	P21796	current study
pYX142-hp18(VDAC)-eGFP	P21796	current study
pYX142-VDAC1	P21796	current study
pYX142-VDAC1Δhp1	P21796	current study
pYX142-VDAC1Δhp18	P21796	current study
pRS426-TPI-Tom20	P35180	[11]
pGEM4-Mcr1	P36060	[12]
C-YC426ADH		[13]
p426ADH-Tom20-YFP(C)	P35180	current study
p426ADH-Mcr1(R4E, R7E)-YFP(C)	P36060	current study
C-YN425ADH		[13]
pYX142-YFP(N)		current study
pYX142-cp(VDAC)-YFP(N)	P21796	current study
pYX142-hp18(VDAC)-YFP(N)	P21796	current study

Supplementary Table 3 | Antibodies used in this study.

Antibody directed against	Dilution	Number
S. cerevisiae (S.c.) Aconitase (purified full-length protein)	1:5000	sc-Aco
S.c. Bmh1 (QQPPAAAEGEAPK)	1:1000	sc-Bmh1
mouse DHFR	1:1000	610697 (BD Biosciences)
S.c. Fis1 (purified protein comprising AA 1-98 fused to MBP)	1:1000	309
GFP	1:5000	11814460001 (Roche)
GFP (β-strand #11, RDHMVLHEYVNAAGIT)	1:2000	(PSL GmbH)
N.c. Porin (SDIAKSANDLLNKDC, antiserum detects S.c. Porin)	1:3000	PorN5
S.c. Tob55 (purified his-tagged full-length protein)	1:2000	321
S.c. Tom20 (CKAESDAVAEANDIDD)	1:1000	sc-Tom20
S.c. Tom22 (CMVELTEIKDDVV)	1:1000	YTom22-N11
S.c. Tom40 (CADGNPLQALPQL)	1:4000	45300
S.c. Tom70 (purified protein comprising AA 33-616)	1:2000	312
A. thaliana VDAC1	1:5000	AS07 212 (Agrisera)
H. sapiens VDAC1	1 µg/mL	ab15895 (Abcam)

Supplementary References

1 Machettira, A. B. *et al.* The localization of Tic20 proteins in Arabidopsis thaliana is not restricted to the inner envelope membrane of chloroplasts. *Plant Mol. Biol.* **77**, 381-390 (2011).

2 Sommer, M. S. *et al.* Chloroplast Omp85 proteins change orientation during evolution. *Proc. Natl. Acad. Sci. U S A* **108**, 13841-13846 (2011).

3 Ulrich, T., Gross, L. E., Sommer, M. S., Schleiff, E. & Rapaport, D. Chloroplast β-barrel proteins are assembled into the mitochondrial outer membrane in a process that depends on the TOM and TOB complexes. *J. Biol. Chem.* **287**, 27467-27479 (2012).

4 Engl, G., Florian, S., Tranebjærg, L. & Rapaport, D. Alterations in expression levels of deafness dystonia protein 1 affect mitochondrial morphology. *Hum. Mol. Gen.* **21**, 287-299 (2012).

5 Paschen, S. A. *et al.* Evolutionary conservation of biogenesis of β-barrel membrane proteins. *Nature* **426**, 862-866 (2003).

6 Mayer, A., Lill, R. & Neupert, W. Translocation and insertion of precursor proteins into isolated outer membranes of mitochondria. *J. Cell Biol.* **121**, 1233-1243 (1993).

7 Pfanner, N., Muller, H. K., Harmey, M. A. & Neupert, W. Mitochondrial protein import: involvement of the mature part of a cleavable precursor protein in the binding to receptor sites. *EMBO J.* **6**, 3449-3454 (1987).

8 Vestweber, D. & Schatz, G. Point mutations destabilizing a precursor protein enhance its post-translational import into mitochondria. *EMBO J.* **7**, 1147-1151 (1988).

9 Pfanner, N. & Neupert, W. Distinct steps in the import of ADP/ATP carrier into mitochondria. *J. Biol. Chem.* **262**, 7528-7536 (1987).

10 Westermann, B. & Neupert, W. Mitochondria-targeted green fluorescent proteins: convenient tools for the study of organelle biogenesis in *Saccharomyces cerevisiae*. *Yeast* **16**, 1421-1427 (2000).

11 Waizenegger, T., Stan, T., Neupert, W. & Rapaport, D. Signal-anchor domains of proteins of the outer membrane of mitochondria: structural and functional characteristics. *J. Biol. Chem.* **278**, 42064-42071 (2003).

12 Meineke, B. *et al.* The outer membrane form of the mitochondrial protein Mcr1 follows a TOM-independent membrane insertion pathway. *FEBS Lett.* **582**, 855-860 (2008).

13 Skarp, K. P., Zhao, X., Weber, M. & Jantti, J. Use of bimolecular fluorescence complementation in yeast Saccharomyces cerevisiae. *Methods Mol. Biol.* **457**, 165-175 (2008).
